# Regulation of High-Affinity Iron Acquisition, Including Acquisition Mediated by the Iron Permease FtrA, Is Coordinated by AtrR, SrbA, and SreA in Aspergillus fumigatus

**DOI:** 10.1128/mbio.00757-23

**Published:** 2023-04-24

**Authors:** Annie Yap, Ricarda Volz, Sanjoy Paul, W. Scott Moye-Rowley, Hubertus Haas

**Affiliations:** a Institute of Molecular Biology, Biocenter, Medical University of Innsbruck, Innsbruck, Austria; b Department of Molecular Physiology and Biophysics, Carver College of Medicine, University of Iowa, Iowa City, Iowa, USA; Karlsruhe Institute of Technology

**Keywords:** fungi, molds, *Aspergillus fumigatus*, iron, siderophore, regulation, transcription factor, siderophores

## Abstract

Iron acquisition is crucial for virulence of the human pathogen Aspergillus fumigatus. Previous studies indicated that this mold regulates iron uptake via both siderophores and reductive iron assimilation by the GATA factor SreA and the SREBP regulator SrbA. Here, characterization of loss of function as well as hyperactive alleles revealed that transcriptional activation of iron uptake depends additionally on the Zn_2_Cys_6_ regulator AtrR, most likely via cooperation with SrbA. Mutational analysis of the promoter of the iron permease-encoding *ftrA* gene identified a 210-bp sequence, which is both essential and sufficient to impart iron regulation. Further studies located functional sequences, densely packed within 75 bp, that largely resemble binding motifs for SrbA, SreA, and AtrR. The latter, confirmed by chromatin immunoprecipitation (ChIP) analysis, is the first one not fully matching the 5′-CGGN_12_CCG-3′ consensus sequence. The results presented here emphasize for the first time the direct involvement of SrbA, AtrR, and SreA in iron regulation. The essential role of both AtrR and SrbA in activation of iron acquisition underlines the coordination of iron homeostasis with biosynthesis of ergosterol and heme as well as adaptation to hypoxia. The rationale is most likely the iron dependence of these pathways along with the enzymatic link of biosynthesis of ergosterol and siderophores.

## INTRODUCTION

The versatile lifestyle of the saprobic mold Aspergillus fumigatus allows its survival in diverse environments, including mammalian hosts. This feature makes this fungal species the primary cause of invasive pulmonary aspergillosis, particularly in immunocompromised patients (1, 2). Owing to the limited availability of certain nutrients in such niches, sophisticated nutrient acquisition strategies are essential for virulence of A. fumigatus. Among them, the uptake of the redox metal iron is fundamental for the survival and virulence of A. fumigatus (2). Due to its redox potential, iron or iron-containing cofactors are involved in numerous cellular processes, including the TCA cycle, oxidative phosphorylation, oxidative stress detoxification, cytochrome P450 enzymes, biosynthesis of amino acids, nucleotides, and sterols, and DNA repair and replication (2, 3). However, iron excess is toxic as it catalyzes the formation of reactive oxygen species (3). Consequently, cellular iron homeostasis is based on fine-tuned regulation of uptake, use, and storage of iron, particularly as fungal species appear to lack cellular iron export strategies (2).

A. fumigatus employs two high-affinity iron uptake systems, reductive iron assimilation (RIA) and siderophore-mediated iron acquisition (SIA). RIA starts with extracellular reduction of ferric to ferrous iron by plasma membrane-localized metalloreductases, such as FreB (4, 5). This is followed by reoxidation and coupled cellular import of iron by a membrane-localized protein complex consisting of the multicopper oxidoreductase FetC and the ferric-iron transporter FtrA. SIA starts with the synthesis of low-molecular-mass ferric-iron chelators, termed siderophores. A. fumigatus secretes two siderophores, triacetylfusarinine C (TAFC) and fusarinine C (FsC), to sequester environmental iron (6). Furthermore, it utilizes two siderophores, ferricrocin (FC) and hydroxyferricrocin, for intracellular transport and storage of iron in hyphae and conidia, respectively. The biosynthesis of these siderophores requires several enzymes and cellular compartments, with the first dedicated enzymatic step being hydroxylation of ornithine by SidA (6, 7). Subsequently, the pathways for biosynthesis of extra- and intracellular siderophores splits due to transfer of different acyl groups to *N*^5^-hydroxyornithine, acetyl for intracellular siderophores and anhydromevalonyl for extracellular siderophores (8, 9). FsC and FC are then assembled by nonribosomal peptide synthetases; TAFC is derived by triple *N*^2^-acetylation of FsC. After secretion and chelation of iron, the siderophore-iron complexes are taken up by specific transporters. A. fumigatus employs four transporters for uptake of different siderophore types (10–12). Defects in siderophore biosynthesis or uptake, such as inactivation of SidA or of the TAFC importer MirB, attenuate virulence of A. fumigatus in murine aspergillosis models, emphasizing the importance of TAFC and SIA-mediated iron acquisition for fungal virulence (8, 9, 12, 13).

Maintenance of iron homeostasis requires sophisticated regulation involving several transcription factors (TFs) (2). A. fumigatus employs two iron-sensing TFs termed SreA and HapX (14, 15). SreA is a GATA-type TF that represses both RIA and SIA during iron sufficiency to avoid iron overload (16). HapX is a bZIP (basic leucine zipper domain)-type TF that cooperates with the CCAAT binding complex (CBC) to repress iron-consuming pathways during iron limitation and to activate iron-consuming pathways, such as vacuolar iron storage mediated by the vacuolar transporter CccA during iron excess (17–19). HapX but not SreA has been shown to be crucial for virulence of A. fumigatus in murine aspergillosis models, which underlines iron limitation in the host niche (20). Furthermore, the basic helix-loop-helix (bHLH) TF SrbA, a member of the sterol regulatory element binding protein (SREBP), was shown to be required for transcriptional activation of RIA and SIA during iron limitation, independent of SreA and HapX (20). In addition to its role in iron metabolism, SrbA plays a crucial role in sterol feedback regulation and adaptation to hypoxic conditions and consequently in resistance against triazole drugs and virulence (21).

More recently, inactivation of the Zn_2_Cys_6_-type TF AtrR in A. fumigatus was found to largely phenocopy lack of SrbA with regard to triazole resistance and growth under hypoxic conditions, indicating cooperation of these two TFs (22, 23); i.e., inactivation of either AtrR or SrbA results in triazole susceptibility due to their role in transcriptional activation of ergosterol biosynthetic genes, blocks growth under hypoxic conditions, and attenuates virulence in mouse models of pulmonary aspergillosis. In agreement with this, promoter occupation by both AtrR and SrbA was found to be required for transcriptional activation of *cyp51A*, which encodes the target of triazole drugs (22–27). However, transcriptional activation of *abcG1* (also known as *cdr1B*), which encodes an ATP-binding cassette (ABC) transporter influencing triazole resistance, was found to be controlled by AtrR but not SrbA (24, 25), indicating that the cooperation of these two TFs is not required for all target genes. It is noteworthy that overexpression of *atrR* using the *hspA* (encoding heat shock protein 7) promoter or C-terminal tagging with 3 hemagglutinin epitopes (3× HA tag) leads to hyperactivation of AtrR, resulting in increased expression of ergosterol biosynthetic genes and increased triazole resistance (25).

Based on the role of SrbA in regulation of iron acquisition and its cooperation with AtrR in the control of ergosterol biosynthesis, we investigated here the potential role of AtrR in regulation of iron metabolism and its interplay with SrbA and SreA.

## RESULTS

### Loss of AtrR causes an iron-dependent growth defect in A. fumigatus.

**FIG 1:**
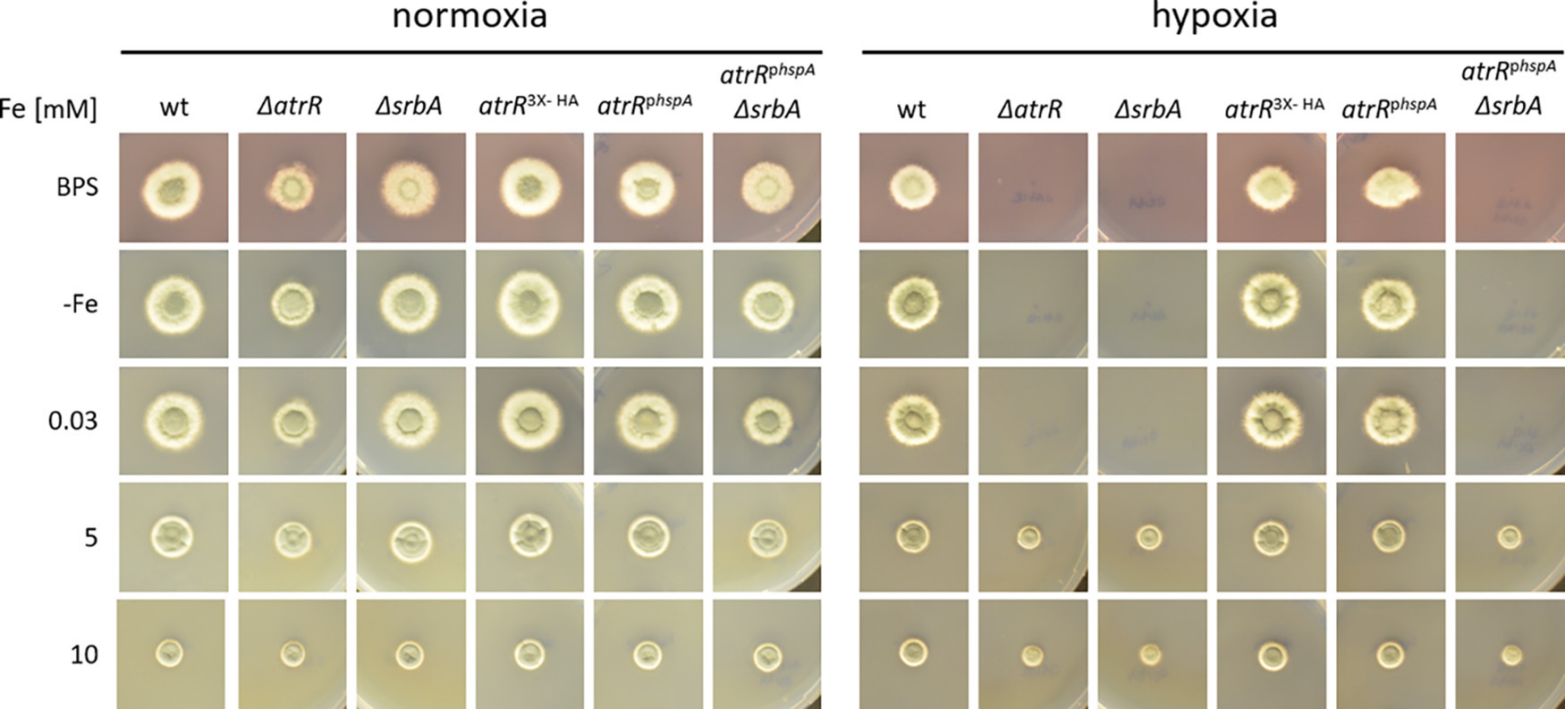
Loss of AtrR causes an iron-dependent growth defect in A. fumigatus. To determine the role of AtrR in iron adaptation, 1 × 10^4^ conidia of wt (Afs35), Δ*atrR*, Δ*srbA*, *atrR^3X-HA^*, and *atrR*^p^*^hpsA^ ΔsrbA* strains were point inoculated on AMM agar plates containing different iron concentrations (−Fe, without iron addition) or the iron chelator BPS (0.2 mM). The plates were incubated at 37°C for 48 h under normoxic or hypoxic (0.2% O_2_) conditions.

To investigate the potential role of AtrR in maintenance of iron homeostasis and to compare its role with that of SrbA, the growth of previously described A. fumigatus strains (24, 25) with the mutations Δ*atrR* (lacking AtrR), AtrR-hyperactive *atrR*^3×HA^ (AtrR C-terminally tagged with the hemagglutinin peptide), *atrR*^p^*^hspA^* (overexpressing *atrR* under the control of the *hspA* promoter), Δ*srbA* (lacking *srbA*), and *atrR*^p^*^hspA^ ΔsrbA* (lacking SrbA and overexpressing *atrR* under the control of the *hspA* promoter) and the respective wild type (wt) were grown on Aspergillus minimal medium (AMM) plates reflecting different iron availability under both normoxic and hypoxic conditions (Fig. 1). Under normoxia, the Δ*atrR* strain exhibited reduced growth under iron-limiting conditions, particularly in the presence of the ferrous iron-specific chelator bathophenanthroline disulfonate (BPS), which blocks reductive iron assimilation (13). The two strains with hyperactive AtrR, the *atrR*^3×HA^ and *atrR*^p^*^hspA^* mutants, showed wt-like growth irrespective of iron availability. However, the hyperactive *atrR*^p^*^hspA^* allele was not able to cure the growth defect caused by loss of SrbA (*atrR*^p^*^hspA^ ΔsrbA* strain) during iron starvation in the presence of BPS (Fig. 1). Notably, a similar interaction of *srbA* and *atrR* alleles was previously observed with respect to triazole resistance and growth in hypoxia (25).

As previously reported (21, 23), lack of either AtrR or SrbA impairs growth under hypoxic conditions (Fig. 1). Remarkably, the growth defect of the Δ*atrR* mutant was partially cured by supplementation with high iron concentrations, such as 5 mM or 10 mM (Fig. 1), as shown previously and confirmed here for the Δ*srbA* strain (28). As observed for iron limitation in normoxia, *atrR*^3×HA^ and *atrR*^p^*^hspA^* strains displayed wt-like growth under all conditions tested, and the hyperactive *atrR*^p^*^hspA^* allele was not able to cure the growth defect caused by loss of SrbA (*hspA*^p^*^hspA^ ΔsrbA* strain) in hypoxia (Fig. 1). Taken together, these data indicated a role of AtrR in iron homeostasis similar to and most likely in cooperation with SrbA.

### AtrR is crucial for submersed growth and siderophore production during iron limitation.

**FIG 2:**
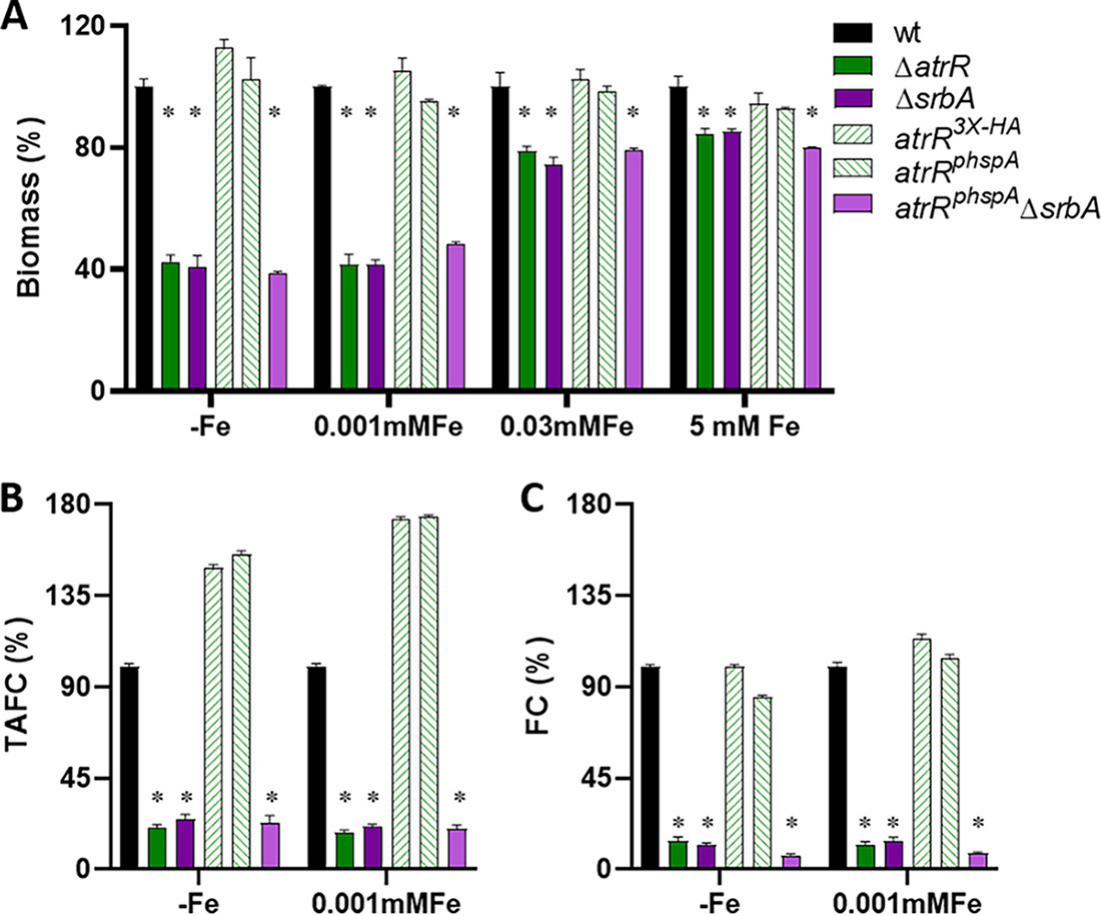
Similar to SrbA, AtrR plays a crucial role in liquid growth and siderophore biosynthesis during iron starvation. (A) For determination of biomass production, 1 × 10^6^ conidia/mL were inoculated in 100 mL liquid AMM containing different iron concentrations or lacking iron supplementation (−Fe) and shaken at 200 rpm for 24 h at 37°C. Biomass was normalized to the wt grown under the same condition (0.194 ± 0.01 for −Fe, 0.556 ± 0 with 0.001 mM Fe, 0.659 ± 0.03 with 0.03 mM Fe, and 0.655 ± 0.02 with 5 mM Fe). Production of extracellular TAFC (B) and intracellular FC (C) was quantified after growth for 24 h at 37°C in −Fe and 0.001 mM Fe. Siderophore production was normalized to the respective biomass and subsequently to that of the wt grown under the same condition. The values are means and standard deviations (SD) for biological triplicates. *, *P* ≤ 0.001 relative to the wt according to two-way analysis of variance (ANOVA).

As a next step, the growth in liquid culture with different iron availability and siderophore production of the above-described strains was analyzed (Fig. 2A). Compared to the wt, lack of AtrR (Δ*atrR*) decreased the biomass production with 5 mM, 0.03 mM, and 0.001 mM iron and without iron supplementation to 84%, 79%, 42%, and 35%, respectively, which was similar to the impact of lack of SrbA (Δ*srbA*): 85%, 74%, 41%, and 42%, respectively (Fig. 2A). Biomass formation of *atrR*^3×HA^ and *atrR*^p^*^hspA^* strains was wt-like under all conditions tested, and the hyperactive *atrR*^p^*^hspA^* allele was not able to cure the growth defect caused by loss of SrbA (*hspA*^p^*^hspA^ ΔsrbA* strain) (Fig. 2A).

As SrbA was found to be required for activation of high-affinity iron uptake, including siderophore-mediated iron acquisition (28), we analyzed production of extracellular TAFC and intracellular FC by the different mutant strains during iron limitation. Similar to the Δ*srbA* mutant, which displayed a decrease of TAFC production to 22% with 0.001 mM iron and 26% without iron supplementation compared to the wt, the Δ*atrR* mutant showed a reduction to 18% in 0.001 mM iron and 20% without iron supplementation (Fig. 2B). Notably, TAFC production by the two strains carrying the hyperactive *atrR* alleles, *atrR*^3×HA^ and *atrR*^p^*^hspA^*, was 1.5-fold and 1.6-fold increased without iron supplementation as well as 1.7-fold increased with 0.001 mM iron compared to that by the wt (Fig. 2B). Overexpression of *atrR* did not impact TAFC production in the absence of SrbA (*hspA*^p^*^hspA^ ΔsrbA* strain) (Fig. 2B). During iron sufficiency (0.03 mM), TAFC production was not detected in any of these strains (data not shown), as reported previously for the wt and Δ*srbA* strains (28).

Lack of AtrR also decreased intracellular accumulation of FC to 12% with 0.001 mM iron and 14% without iron supplementation compared to the wt, which matched the effect of loss of SrbA (Fig. 2C). The hyperactive AtrR alleles *atrR*^3×HA^ and *atrR*^p^*^hspA^* did not affect FC accumulation, and overexpression of *atrR* did not compensate for the negative effect of SrbA deficiency (*atrR*^p^*^hspA^ ΔsrbA* strain) on FC accumulation (Fig. 2C).

Taken together, these data indicate that adaptation to iron limitation with respect to growth and siderophore production depends similarly on AtrR and SrbA. The cooperation of these two TFs is emphasized by the fact that overexpression of *atrR* was not able to compensate for the defect caused by lack of SrbA. Nevertheless, the hyperactive AtrR alleles were able to increase extracellular siderophore production.

### AtrR is essential for transcriptional activation of genes involved in iron acquisition as well as biosynthesis of heme and ergosterol.

**FIG 3:**
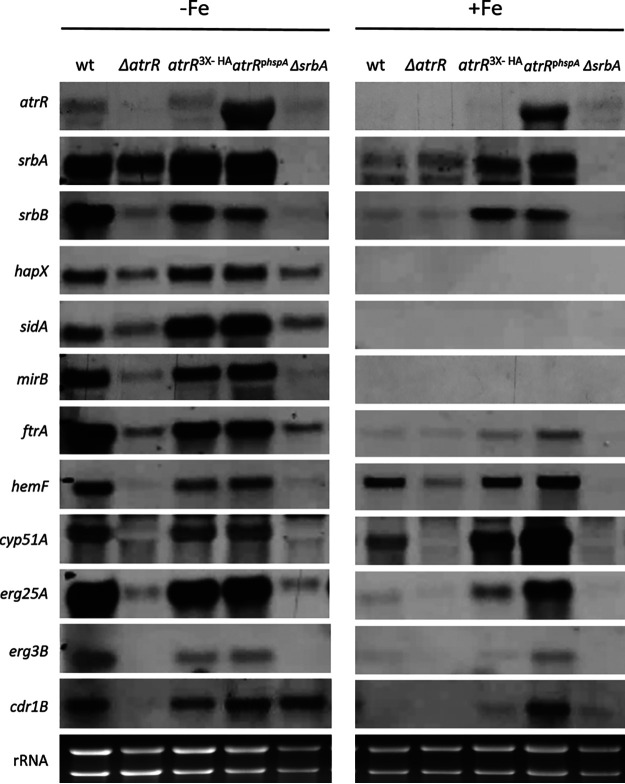
Similar to SrbA, AtrR is crucial for transcriptional activation of genes involved in adaptation to iron starvation as well as biosynthesis of heme and ergosterol. For Northern blot analysis, total RNA was isolated from A. fumigatus strains grown during iron limitation (−Fe) or iron sufficiency (+Fe) for 24 h at 37°C. Ethidium bromide-stained rRNA is shown as a control for loading and quality of RNA. Genes are described in the text and Table S2.

To further investigate the role of AtrR in comparison to SrbA with respect to transcriptional regulation of iron metabolism, we conducted Northern blot analyses of several key genes involved in adaptation to iron starvation as well as of genes previously reported to be regulated by these two TFs under conditions of iron starvation and iron sufficiency (Fig. 3). Notably, iron starvation increased the transcript levels of *atrR*, *srbA*, and *srbB*, which encodes another SREBP-type TF that is involved in regulation of biosynthesis of ergosterol and heme (21, 28). Northern blot analysis also confirmed overexpression of *atrR* in the *atrR*^p^*^hspA^* strain (25). Particularly during iron starvation, lack of AtrR decreased expression of *srbA* and vice versa, emphasizing the interdependence of the encoded TFs, and both were required for transcriptional activation of *srbB*. Similar to lack of SrbA, as shown previously and confirmed here (28), inactivation of AtrR during iron starvation reduced transcript levels of the following genes, which were shown previously to be induced by iron starvation and to be required for adaptation to iron starvation: *hapX* (encoding an iron-sensing TF required for repression of iron-dependent pathways), *sidA* (encoding ornithine hydroxylase, which is essential for biosynthesis of extra- and intracellular siderophores), *mirB* (encoding the TAFC importer), and *ftrA* (encoding the iron permease that is essential for RIA). Notably, the hyperactive AtrR alleles *atrR*^3×HA^ and *atrR*^p^*^hspA^* increased expression of *sidA* compared to the wt (Fig. 3), which is in line with increased TAFC production found in the respective mutant strains named above (Fig. 2B). In contrast, overexpression of *atrR* did not impact the defective transcriptional activation caused by SrbA deficiency (*hspA*^p^*^hspA^ ΔsrbA* strain). Taken together, these data clearly indicate a role of AtrR in transcriptional activation of the iron starvation response in cooperation with SrbA and provide an explanation for defective growth and siderophore biosynthesis under iron starvation caused by lack of AtrR.

10.1128/mbio.00757-23.2 TABLE S2 Oligonucleotides used to generate digoxigenin-labeled probes for Northern analysis. Download Table S2, PDF file, 0.3 MB. Copyright © 2023 Yap et al. 2023 Yap et al. https://creativecommons.org/licenses/by/4.0/ This content is distributed under the terms of the Creative Commons Attribution 4.0 International license.

As shown previously (25, 28), both SrbA and AtrR were crucial for transcriptional activation of the heme biosynthetic gene *hemF* and the ergosterol biosynthetic genes *cyp51A*, *erg25A*, and *erg3B*, while only AtrR and not SrbA was important for expression of ABC transporter-encoding *cdr1B* during both iron starvation and sufficiency. Furthermore, the two hyperactive AtrR alleles, in particular *atrR*^p^*^hspA^*, caused increased expression of *cyp51A*, *erg25B*, and *cdr1B* during iron sufficiency compared to the wt (Fig. 3). These data confirm the previously described mutant strain characteristics.

Similar to the results of growth assays (Fig. 1), overexpression of *atrR* was not able to compensate for the defects caused by SrbA deficiency (*hspA*^p^*^hspA^ ΔsrbA* strain) at the level of transcription (see Fig. S1 in the supplemental material), which again indicates that SrbA and AtrR cooperate in transcriptional activation of genes involved in SIA and RIA.

10.1128/mbio.00757-23.5 FIG S1 Hyperactive *atrR* alleles cannot compensate for defective gene regulation caused by lack of *srbA*. For Northern blot analysis, total RNA was isolated from A. fumigatus strains grown during iron limitation (−Fe) or iron sufficiency (+Fe) for 24 h at 37°C. Ethidium bromide-stained rRNA is shown as a control for loading and quality of RNA. Genes are described in the text and Table S2. Download FIG S1, TIF file, 0.6 MB. Copyright © 2023 Yap et al. 2023 Yap et al. https://creativecommons.org/licenses/by/4.0/ This content is distributed under the terms of the Creative Commons Attribution 4.0 International license.

### Overexpression of *srbA* does not compensate for the lack of AtrR.

As shown above, hyperactive AtrR alleles are not able to compensate for the lack of SrbA. To test the inverse, *srbA* was expressed under the control of the xylose-inducible *xylP* promoter (29, 30) integrated in a single copy into the *fcyB* locus and thus allowing selection marker-independent genomic integration (31) in A. fumigatus wt, Δ*srbA* and Δ*atrR* strains, yielding *srbA*^p*xylp*^, *srbA*^p*xylp*^Δ*srbA*, and *srbA*^p*xylp*^Δ*atrR* strains. Notably, the *srbA*^p^*^xylP^ and srbA*^p^*^xylP^* Δ*atrR* strains also possess the endogenous *srbA*. Overexpression of *srbA* cured the lack of *srbA* (*srbA*^p^*^pxylP^* Δ*srbA*) under inducing (1% glucose–0.1% xylose as the carbon source) but not under repressing (1% glucose as the carbon source) conditions with respect to growth under hypoxia (Fig. S2), production of biomass and siderophores under iron limitation (Fig. S3) as well as transcriptional activation of genes involved in siderophore biosynthesis (*sidA*), siderophore uptake (*mirB*), reductive iron assimilation (*ftrA*) and ergosterol biosynthesis (*erg25*) (Fig. S4). These results prove the functionality of the *srbA*^p^*^xylP^* allele. Despite high *srbA* transcript levels (Fig. S4), however, *srbA* overexpression did not cure any defects caused by lack of AtrR (Fig. S2 to S4). These data underline that transcriptional activation of genes involved in SIA, RIA, and ergosterol biosynthesis require cooperation of SrbA and AtrR.

10.1128/mbio.00757-23.6 FIG S2 Overexpression of *srbA* cannot suppress the iron-dependent growth defect of the Δ*atrR* strain. Conidia (1 × 10^4^) of each A. fumigatus strain were point inoculated on AMM agar plates without iron addition (−Fe). The plates were incubated at 37°C for 48 h under normoxic or hypoxic (0.2% O_2_) conditions. Download FIG S2, TIF file, 1.7 MB. Copyright © 2023 Yap et al. 2023 Yap et al. https://creativecommons.org/licenses/by/4.0/ This content is distributed under the terms of the Creative Commons Attribution 4.0 International license.

10.1128/mbio.00757-23.7 FIG S3 Overexpression of *srbA* cannot compensate the growth and siderophore production defect of Δ*atrR* in liquid culture. (A) For determination of biomass production, 1 × 10^6^ conidia/mL was inoculated in 100 mL liquid AMM lacking iron supplementation (−Fe) and shaken at 200 rpm for 24 h at 37°C. Biomass was normalized to the wt grown under the same conditions, 1% Glc (0.128 ± 0.01) and 1% Glc–0.1% Xyl (0.127 ± 0.00). Production of extracellular TAFC (B) and intracellular FC (C) was quantified after growth for 24 h at 37°C under −Fe conditions. Siderophore production was normalized to the respective biomass and subsequently to that of the wt grown under the same condition. Values are means and SD for biological triplicates. *, *P* ≤ 0.001 according to two-way ANOVA. Download FIG S3, TIF file, 0.7 MB. Copyright © 2023 Yap et al. 2023 Yap et al. https://creativecommons.org/licenses/by/4.0/ This content is distributed under the terms of the Creative Commons Attribution 4.0 International license.

10.1128/mbio.00757-23.8 FIG S4 Overexpression of *srbA* cannot compensate for defective gene regulation caused by lack of *atrR*. For Northern blot analysis, total RNA was isolated from A. fumigatus strains grown during iron limitation (−Fe) in the presence of 1% Glc or 1% Glc–0.1% Xyl for 24 h at 37°C. Ethidium bromide-stained rRNA is shown as control for loading and quality of RNA. Genes are described in the text and Table S2. Download FIG S4, TIF file, 1.1 MB. Copyright © 2023 Yap et al. 2023 Yap et al. https://creativecommons.org/licenses/by/4.0/ This content is distributed under the terms of the Creative Commons Attribution 4.0 International license.

### Reporter gene-mediated p*ftrA* truncation analysis identified regulatory regions.

**FIG 4:**
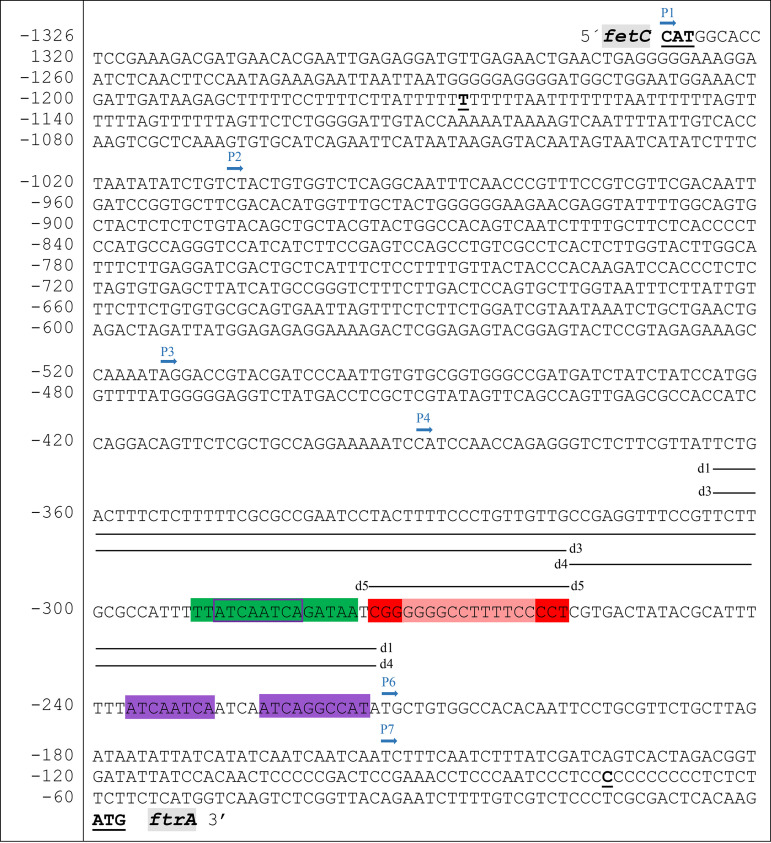
Nucleotide sequence of the bidirectional intergenic region of *ftrA* and *fetC*. The transcription start sites of both *ftrA* and *fetC* are in bold and underlined. The putative DNA binding motifs of different TFs are highlighted: SreA (5′-ATCWGATAA-3′ combined with a preceding GATAA motif on the complementary strand) in green, SrbA (5′-TCANNCCA-3′) in purple, AtrR (5′-CGG-X_12_-CCG-3′) in red, and a putative SrbA binding motif overlapping the SreA motif in purple. The start sites of the different truncations and the deletions are indicated above the sequence by blue arrows and black lines, respectively. The nucleotide numbering refers to the *ftrA* TSS.

The role of SreA, SrbA, and now AtrR in regulation of genes involved in iron acquisition is based mainly on gene expression profiling of mutants lacking these TFs, and consequently, indirect effects cannot be excluded. Therefore, we employed mutational promoter analysis of the *ftrA* promoter (p*ftrA*) here for the following reasons: (i) *ftrA* is essential for RIA and highly conserved in siderophore-producing and non-siderophore-producing fungal species (6); (ii) *ftrA* has a defined promoter region, as it shares a bidirectional promoter with the *fetC* gene, encoding the ferroxidase involved in RIA (13); (iii) based on expression profiling of mutants lacking the respective TF, *ftrA* has previously been found to be regulated by SreA and SrbA and here by AtrR (16, 25, 28); and (iv) global chromatin immunoprecipitation DNA sequencing (ChIP-seq) analyses previously indicated binding of SrbA and AtrR to the 5′ upstream region of *ftrA* (21, 25, 28). The p*ftrA* sequence is displayed in Fig. 4.

**FIG 5:**
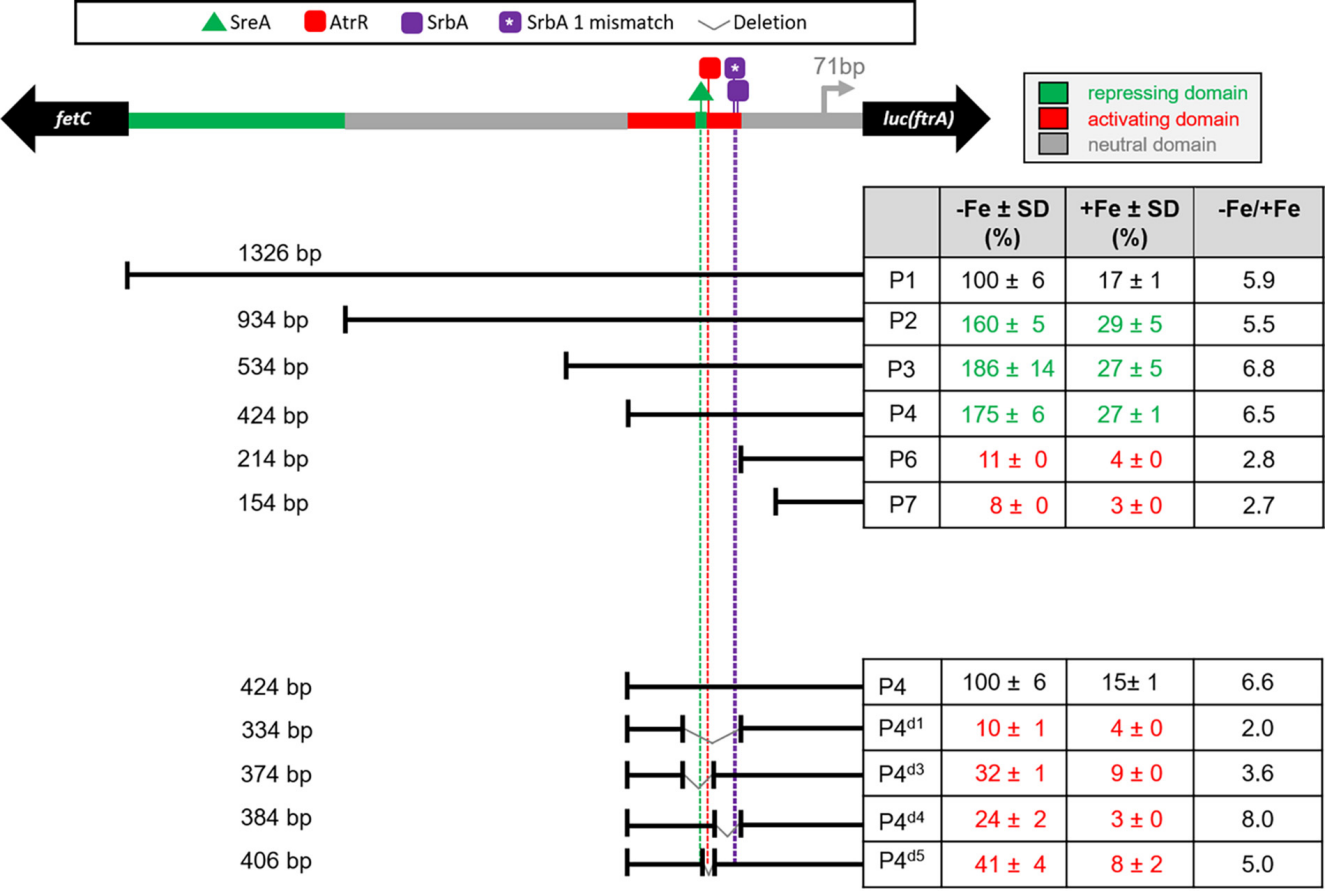
Truncations and deletion of p*ftrA* using *luc* as a reporter for promoter activity identified functional regions. Promoter activity was measured during iron limitation (−Fe) and sufficiency (+Fe) as described in Materials and Methods. The promoter activities are means and SD for biological triplicates normalized to P1 (top) or P4 (bottom). The −Fe/+Fe column displays the ratio of promoter activity under −Fe and +Fe iron conditions of the different p*ftrA* versions. Values in red and green indicate decreased and increased promoter activity relative to P1 or P4, respectively. The raw data are shown in Table S4A.

To characterize p*ftrA* and to define regulatory regions, 11 p*ftrA* promoter versions containing truncations (termed P2 to P7) or deletions (termed P4^d1^ to P4^d5^) were fused with the luciferase-encoding *luc* gene from Photinus pyralis as a reporter for promoter activity (Fig. 5). The promoter constructs were integrated in single copies into the *fcyB* locus of A. fumigatus strain A1160+, allowing selection marker-independent genomic integration (31). Notably, as indicated by growth assays (Fig. S5), the identified role of AtrR and SrbA in iron regulation is conserved in A. fumigatus strains A1160+ and AfS35. To facilitate comparison, promoter activity of all promoter versions was normalized to P1 and P4, respectively (Fig. 5). As expected from transcriptional analysis (Fig. 3), promoter activity of the p*ftrA* version containing the entire intergenic region, P1 (1,326 bp), was higher during iron limitation than iron sufficiency; in fact, it was 5.9-fold higher. Truncation of the p*ftrA* to 934 bp in P2 increased the promoter activity 1.6-fold compared to that of P1, indicating that the 392-bp upstream region contains repressing elements. Further truncation to 534 bp in P3 or 424 bp in P4 had only a marginal impact on promoter activity. In contrast, further truncations to 214 bp in P6 and 154 bp in P7 decreased promoter activity to 11% and 8%, respectively. These results indicated that the major activation of p*ftrA* is mediated by the 210-bp region between 424 bp and 214 bp upstream of the *ftrA* translation start site (TSS), termed p*ftrA*^210^. Notably, the ratio of promoter activity during iron starvation compared to iron sufficiency (−Fe/+Fe) decreased in P6 to 2.8, indicating that p*ftrA*^210^ mediates regulation in a manner that is dependent on iron availability.

10.1128/mbio.00757-23.9 FIG S5 The effects caused by inactivation or mutation of *atrR* or *srbA* in A. fumigatus A1160+ are similar to those in A. fumigatus Afs35 (Fig. 1). Conidia (1 × 10^4^) of A. fumigatus A1160+ background strains were point inoculated on AMM agar plates containing different iron concentrations (−Fe, without iron addition) or the iron chelator BPS (0.2 mM). The plates were incubated at 37°C for 48 h under normoxic or hypoxic (0.2% O_2_) conditions. Download FIG S5, TIF file, 0.7 MB. Copyright © 2023 Yap et al. 2023 Yap et al. https://creativecommons.org/licenses/by/4.0/ This content is distributed under the terms of the Creative Commons Attribution 4.0 International license.

10.1128/mbio.00757-23.4 TABLE S4 Raw bioluminescence of A. fumigatus *luc* reporter strains. Data are from three biological replicates measured simultaneously in experiments A, B, and C during iron starvation (−Fe) and sufficiency (+Fe). Download Table S4, PDF file, 0.3 MB. Copyright © 2023 Yap et al. 2023 Yap et al. https://creativecommons.org/licenses/by/4.0/ This content is distributed under the terms of the Creative Commons Attribution 4.0 International license.

### The p*ftrA*^210^ sequence autonomously mediates iron regulation.

**FIG 6:**
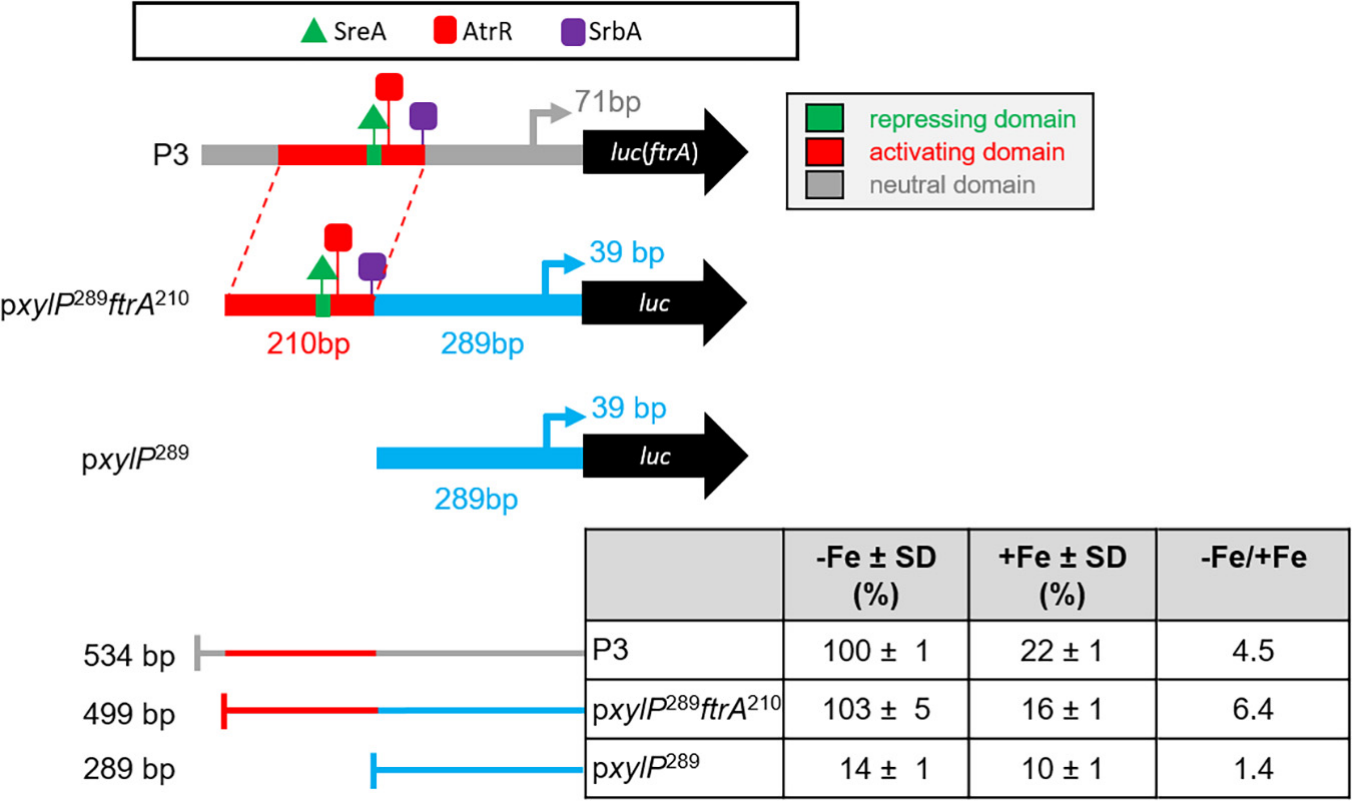
p*ftrA*^210^ mediates that iron regulation when fused with the p*xylP*^289^ minimal promoter. Promoter activity was measured as described in Materials and Methods. Data are means and SD for three biological replicates normalized to P3 grown under −Fe or +Fe conditions. The −Fe/+Fe column displays the ratio of promoter activity under −Fe and +Fe conditions of the different promoters. The raw data are shown in Table S4B.

The results described above indicated that p*ftrA*^210^ contains crucial elements for activation and repression of *ftrA*. To investigate if this sequence is able to operate independently of other p*ftrA* elements, p*ftrA*^210^ was fused with a 289-bp promoter fragment of the p*xylP* promoter (30, 32), termed p*xylP*^289^, which displays low promoter activity during both iron starvation and iron sufficiency (Fig. 6). Remarkably, the hybrid promoter p*xylP*^289^*ftrA*^210^ conferred the same iron regulation as P3, including activation during iron starvation and repression during iron sufficiency. These data strongly indicate that p*ftrA*^210^ contains the major binding motifs for TF mediating transcriptional iron regulation of *ftrA*.

### *In silico* prediction of putative DNA binding motifs for SreA, SrbA, and AtrR.

In the next step, we aimed to identify potential TF recognition motifs in the p*ftrA*^210^ sequence by visual inspection. The GATA-type TF SreA is expected to recognize the motif 5′-(A/C/T)GATA(G/A)-3′, whereby an extended and partial palindromic variation of this motif, 5′-ATCWGATAA-3′, was discovered to be significantly overrepresented in upstream regions of A. fumigatus SreA regulon member genes (16). Indeed, p*ftrA*^210^ contains such a motif, 5′-ATCAGATAA-3′, combined with a preceding 5′-TGATAA-3′ motif located on the complementary strand 217 bp upstream of the TSS (Fig. 4).

A combination of ChIP-seq and MEME (multiple expectation maximizations for motif elicitation) analysis identified 5′-(A/G)TCA(T/C/G)(C/G)CCAC(T/C)-3′ as the binding motif for A. fumigatus SrbA (21, 28), a sequence that is similar to the previously identified SrbA DNA binding motif 5′-ATC(G/A)(T/G)(A/G)(C/T)(G/C)AT-3′, discovered using bioinformatic tools (33). It is noteworthy that several fungal SREBPs were found to bind preferentially to the nonpalindromic 5′-TCANNCCA-3′ motif, with 5′-ATCAGGCCAT-3′ being recognized with high affinity by A. fumigatus SrbA (34). Remarkably, p*ftrA*^210^ contains the exact latter sequence 151 bp upstream of the TSS and two similar versions, 5′-ATCAATCAAT-3′ and 5′-ATCAATCAGA-3′, 159 bp and 211 bp upstream of the TSS (Fig. 4), which deviate by one and two nucleotides, respectively, from the consensus sequence, 5′-TCANNCCA-3′. Notably, 5′-ATCAATCAGA-3′ overlaps the putative SreA binding motif.

AtrR is a Zn_2_Cys_6_ cluster-containing TF shown to recognize the consensus sequence 5′-CGGN_12_CCG-3′, often in close proximity to an SrbA recognition motif, such as in *cyp51A*, while this consensus motif is not sufficient to explain the entire range of target genes with promoters that often contain only the 5′-CCG-3′ half site of this motif (22, 25, 26). The sequence that most closely resembles the AtrR consensus 5′-CGGN_12_CCG-3′ in p*ftrA*^210^ is 5′-CGGGGGGCCTTTTCCCCT-3′, 197 bp upstream of the TSS, in close proximity to the putative SreA and SrbA binding sites (Fig. 4).

### Reporter gene-mediated p*ftrA* deletion and mutation analysis identified TF binding sites.

To validate the *in silico*-predicted DNA binding motifs, deletion analysis in p*ftrA* version P4 within the p*ftrA*^210^ region using *luc* as a reporter gene was carried out (Fig. 4 and 5). Deletion of a 90-bp region in P4^d1^ (230 to 140 bp upstream of the TSS) containing all predicted binding motifs for SreA, AtrR, and SrbA decreased the promoter activity to 10% of full-length P4 during iron starvation and decreased the −Fe/+Fe ratio of promoter activity from 6.0 to 2.2 (Fig. 5). These results indicate that this region indeed contains binding domains required for activation during iron starvation and repression during iron sufficiency. Deletion of only the 5′-positioned 40 bp of this 90-bp region in P4^d3^ resulted in the removal of the putative SreA- and AtrR-binding motifs, reduced the promoter activity to only 32% during iron starvation, and yielded a −Fe/+Fe ratio of promoter activity of 3.6 (Fig. 5). In comparison, deletion of the 3′-positioned 50 bp in P4d^4^ containing the major putative SrbA binding motifs reduced the promoter activity to 24% during iron starvation and showed a −Fe/+Fe ratio of promoter activity of 8.0 (Fig. 5). Deletion of only the predicted AtrR binding motif in P4^d5^ reduced the promoter activity to 41% during iron starvation and resulted in a −Fe/+Fe ratio of promoter activity of 5.0 (Fig. 5). Taken together, these data are in agreement with the prediction of the TF binding sites, with deletion of AtrR- or SrbA-binding motifs decreasing promoter activity, particularly during both iron starvation and sufficiency, as well as deletion of the SreA-binding motifs reducing the −Fe/+Fe ratio of promoter activity.

**FIG 7:**
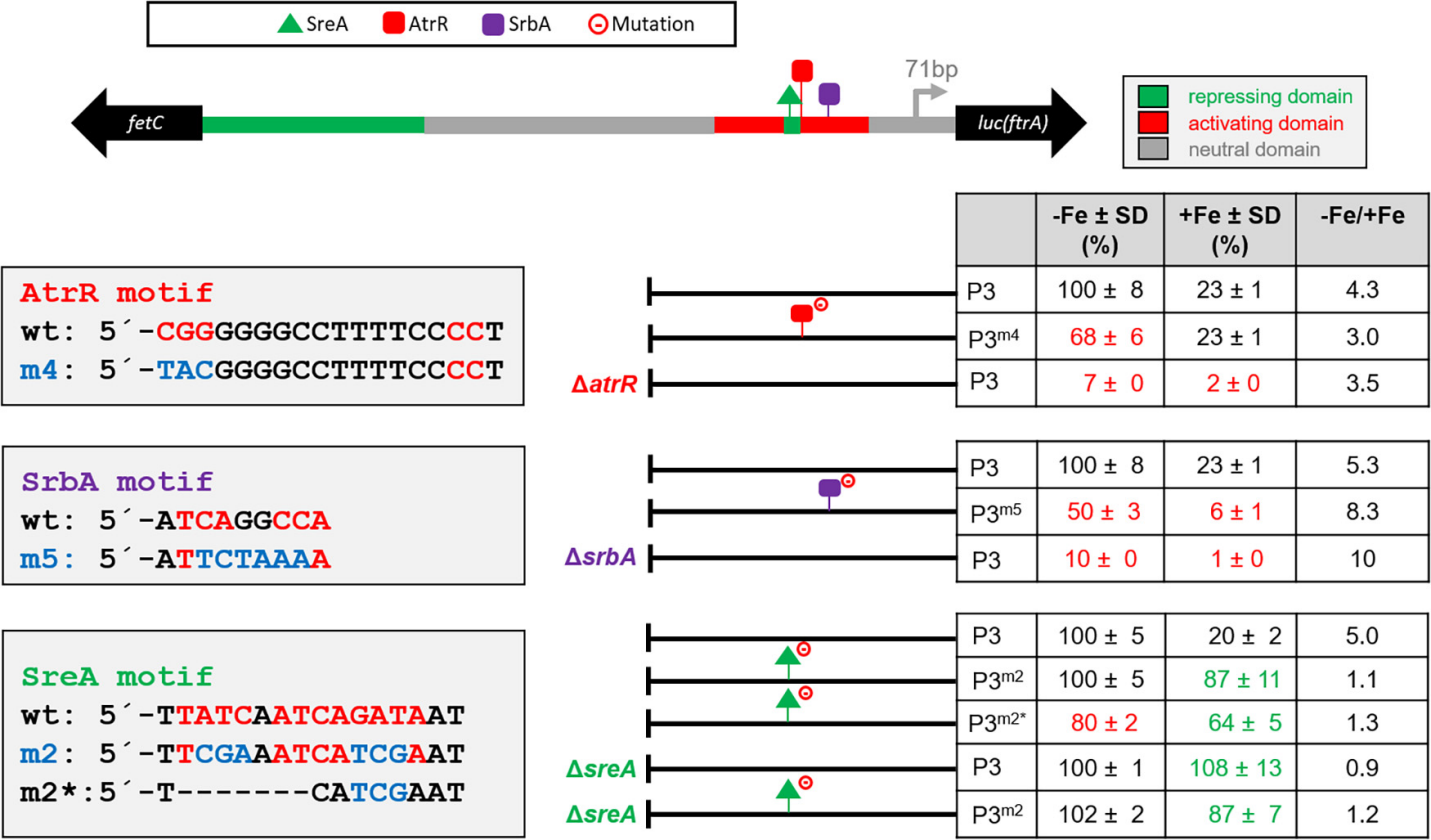
Site-directed mutagenesis identified regulatory motifs in p*ftrA* using *luc* as the reporter gene. Promoter activity was measured as described in Materials and Methods. Data are means and SD for three biological replicates normalized to P3 grown under −Fe or +Fe conditions. The −Fe/+Fe column displays the ratios of promoter activity under −Fe and +Fe iron conditions of the different p*ftrA* versions. Values in red and green indicate decreased and increased promoter activity relative to P3, respectively. The raw data are shown in Table S4C.

To further characterize the individual DNA binding motif, we mutated the *in silico*-identified motifs in p*ftrA* version P3 via nucleotide exchanges (Fig. 7). Mutation of the identified AtrR motif in P3^m4^ and SrbA motif in P3^m5^ reduced the promoter activity to 68% and 50%, respectively, compared to P3. Moreover, introduction of P3 in the Δ*atrR* or Δ*srbA* mutant strains resulted in promoter activity of only 7% and 10%, respectively, compared to that of the wt (Fig. 7). Taken together, these data strongly indicate that expression of *ftrA* is directly regulated by AtrA and SrbA, most likely mediated by the investigated motifs.

The mutation of the SreA DNA binding motif in P3^m2^ resulted in derepression of promoter activity during iron sufficiency, with a −Fe/+Fe ratio of 1.1 (Fig. 7), clearly indicating the importance of this motif in repression during iron sufficiency. In agreement with SreA regulating expression of *ftrA* via this motif, both P3 and P3^m2^ showed derepressed promoter activity when introduced into the Δ*sreA* mutant strain. Interestingly, sequence exchange combined with a 7-bp deletion within the putative SreA binding motif in P3^m2^* resulted not only in derepression of promoter activity during iron sufficiency (−Fe/+Fe ratio of 1.3) but also in a 20% decrease of promoter activity during iron starvation (Fig. 7), indicating that this motif might also be involved in transcriptional activation, possibly by SrbA, as a motif similar to the SrbA consensus binding motif overlaps the SreA motif (see above; also see Fig. 4). Taken together, these data demonstrate that SreA directly represses p*ftrA* activity during iron replete condition.

### Single-gene ChIP confirms the AtrR binding motif in p*ftrA*.

**FIG 8:**
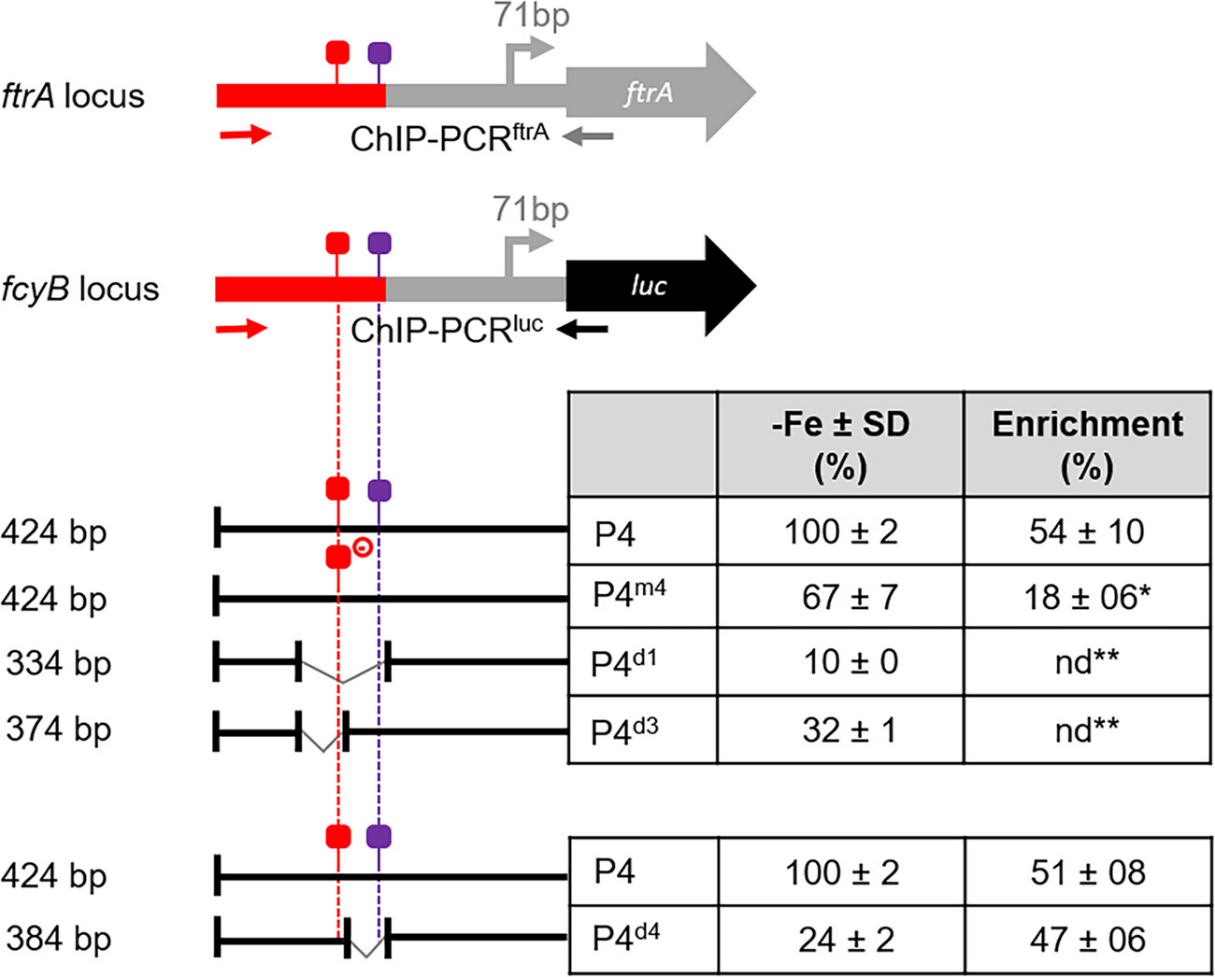
ChIP analysis combined with *luc* promoter activity analysis confirms binding of AtrR to the predicted motif. The *luc* promoter activity was analyzed as described in Fig. 7 and normalized to P4. Data are means and SD of three biological replicates normalized to P4 grown during iron starvation (−Fe). The raw data are shown in Table S4A, ChIP was performed as described in Materials and Methods. Enrichment of AtrR at the P4 p*ftrA* variants at the *fcyB* locus by ChIP during iron starvation (−Fe) was normalized to that at the native p*ftrA*. The values are means and SD representing two independent ChIP reactions with three quantitative real-time PCRs from each ChIP experiment. Statistically significant differences from P4 according to an unpaired two-tailed *t* test are indicated: *, *P* < 0.01; **, *P* < 0.001.

To further elucidate the interaction of AtrR with p*ftrA*, we employed single-gene ChIP to investigate AtrR promoter binding to different P4 versions at the *fcyB* locus compared to endogenous p*ftrA* (Fig. 8). Therefore, four previously described strains with analyzed promoter activity carrying different P4 p*ftrA* versions driving *luc* at the *fcyB* locus (Fig. 5) were grown under iron starvation: P4, P4m^4^ (mutation of the putative AtrR motif), P4^d1^ (90-bp deletion eliminating all predicted putative binding motifs for SreA, AtrR, and SrbA), and P4^d3^ (40-bp deletion resulting in the removal of the putative SreA- and AtrR-binding sites). The immunoprecipitated chromatin was examined for enrichment of the respective fragments of p*ftrA* at the *fcyB* locus as well as the endogenous locus by quantitative PCR (qPCR) discriminated by the respective PCR primers recognizing the different flanking regions (Fig. 8). Promoter enrichment at the p*ftrA* variants integrated at the *fcyB* locus compared to native p*ftrA* was about 52% for P4 and 18% for P4^m4^ (Fig. 8), which contains the AtrR binding motif with the mutated half site (Fig. 7). No enrichment was detected for the P4 versions lacking the AtrR binding motifs, P4^d1^ and P4^d3^ (Fig. 8). These data are in agreement with the promoter activity measured for the different versions: the decreased activity combined with decreased promoter binding by AtrR in P4^m4^ indicates weakened AtrR binding to the mutated motif, while the even further decreased activity combined with absence of AtrR binding is in agreement with the lack of AtrR binding motifs. Taken together, these data support the direct transcriptional activation of *ftrA* by AtrR mediated by the predicted motif.

Deletion of the two putative SrbA binding motifs in P4^d4^ (ATCAATCAATCAATCAGGCCAT; SrbA binding motifs are underlined) (Fig. 4), which reduced promoter activity to about 24% compared to P4 (Fig. 5), did not impact binding of AtrR to p*ftrA* (Fig. 8). These results suggest that AtrR binds to p*ftrA* independently of SrbA promoter occupation.

## DISCUSSION

Adaptation to iron limitation, including the activation of high-affinity iron acquisition, is crucial for the virulence of most pathogens, as the antimicrobial defense of mammalian hosts involves deprivation and sequestration of iron (2, 11). Transcriptional regulation of high-affinity iron acquisition by A. fumigatus involving SIA and RIA has previously been indicated to involve the iron-sensing GATA-type TF SreA for mediating repression during iron sufficiency and the SREBP SrbA for activation during iron starvation (16, 28). The role of SreA appears to be confined to iron regulation, while SrbA is also required for adaption to hypoxia and activation of biosynthesis of ergosterol and heme (21, 28, 35). Consequently, SrbA but not SreA is crucial for triazole resistance and virulence of A. fumigatus (16). More recently, SrbA was indicated to cooperate with the TF AtrR with regard to control of ergosterol biosynthesis, heme biosynthesis, and adaptation to hypoxia but not for regulation of ABC transporter-encoding *abcG1*/*cdr1B*, which is mediated by AtrR alone (24, 25).

Here, growth studies as well as analysis of gene expression and siderophore biosynthesis of mutant strains expressing hyperactive alleles of the Zn_2_Cys_6_-TF AtrR, overexpressing *srbA*, or containing deletions of either AtrR or SrbA revealed that transcriptional activation of both SIA and RIA depends on cooperation of SrbA and AtrR. Moreover, reporter gene-mediated analysis of the promoter of *ftrA*, which encodes an iron permease involved in RIA, identified a 210-bp fragment, termed p*ftrA^210^*, that is essential and sufficient to impart transcriptional iron regulation, i.e., activation during iron starvation and repression during iron sufficiency. The latter was also confirmed by fusion of p*ftrA^210^* with the minimal *xylP* promoter, driving expression of a xylanase in an iron-independent mode. Mutational analysis within p*ftrA^210^* identified functional sequences that resemble the previously described binding motifs for SrbA, SreA, and AtrR, the latter of which was confirmed by ChIP analysis. The identified motifs were (i) the sterol response element (SRE), the recognition site for SrbA 5′-ATCAGGCCAT-3′ resembling 5′-TCANNCCA-3′ (21, 28, 34) and previously found to be recognized with high affinity by A. fumigatus SrbA (34), (ii) 5′-TTATCAATCAGATAA-3′, containing the GATA-type TF consensus motif 5′-(A/C/T)GATA(G/A)-3′ on the complementary strand and the extended, partial palindromic variation 5′-ATCWGATAA-3′ previously found to be enriched in SreA target promoters (16), and (iii) the AtrR response element (ATRE) 5′-CGGGGGGCCTTTTCCCCT-3′, resembling the AtrR consensus found at the *cyp51A* and *abcG1* 5′-CGGN_12_CCG-3′ sequence, with one mismatch (25). Deletion of the entire AtrR motif decreased promoter activity more significantly than mutation of the 5′-CGG-3′ half site, which emphasizes the previously described complex mode of DNA recognition by AtrR, as only a minority of the AtrR target promoters contains the full consensus motif (25). Therewith, the functionally identified ATRE in p*ftrA* is the first functional binding site that does not completely match the consensus sequence. An example of an ATRE that fully matches the consensus motif has been extensively characterized in the *cyp51A* promoter (22, 23, 25–27). Notably, p*ftrA^210^* contained additional variations of the SRE with one or two mismatches, which appeared to also play a role in regulation, as indicated by mutational promoter analysis. Remarkably, one SRE overlapped the putative SreA binding site. Indeed, one of the introduced mutations in the SreA motif caused not only derepression of promoter activity during iron sufficiency, as expected from inactivation of SreA impact, but also decreased activation during iron limitation, which is in agreement with inactivation of a binding motif for a positively acting TF such as SrbA. These results might indicate that competition of SrbA and SreA for binding this sequence might play a role in regulation of *ftrA*. All the identified functional motifs were densely packed within 75 bp, which is reminiscent of the situation in the *cyp51A* promoter, in which four TFs bind, partially overlapping, within 52 bp: SrbA, AtrR, the CCAAT-binding complex (CBC), and HapX (22, 23, 26, 27, 36). So far, the role of SrbA and SreA in regulation of iron acquisition was based mainly on gene expression profiling in mutants lacking these TFs, and consequently, indirect effects could not be excluded. The mutational p*ftrA* promoter analysis presented here illustrates the direct involvement of SrbA, AtrR, and SreA in regulation of *ftrA* expression and iron regulation in general.

The cooperation of SrbA and AtrR in activation of iron acquisition is emphasized by the fact that overexpression of either *atrR* in the Δ*srbA* strain or *srbA* in the Δ*atrR* background failed to compensate for the defects in growth and gene regulation caused by lack of SrbA or AtrR, respectively. Nevertheless, the increased siderophore production concomitant with increased expression of the siderophore-biosynthetic *sidA* gene, caused by *atrR* overexpression or AtrR hyperactivity due to C-terminal tagging with the hemagglutinin peptide (25), in the presence of SrbA, might indicate that AtrR-mediated activation is the limiting factor in the cooperation or that AtrR functions partially independent in the presence of SrbA, or it might be explained by the mutual transcriptional control of AtrR and SrbA as seen in the Northern blot-based expression analysis. The Northern blot data here further illuminate the regulatory link of these TFs, as transcriptional activation of *hapX* and *srbB*, which encodes an SrbA-regulated SREBP-type TF (21, 28), was found to depend on both SrbA and AtrR.

The most likely explanation for the defective adaptation to iron starvation caused by AtrR inactivation observed in the growth assays is the impaired activation of RIA and SIA. As adaption to iron-limiting conditions is crucial for virulence of A. fumigatus (2), the defect in activation of high-affinity iron uptake might at least partially explain the observed avirulence caused by lack of AtrR or SrbA (24).

The essential role of AtrR in transcriptional activation of high-affinity iron acquisition similar to SrbA emphasizes the regulatory coordination of iron uptake with adaptation to hypoxia as well as biosynthesis of ergosterol and heme and consequently resistance against triazole drugs. The rationale is most likely based on the fact that all mentioned pathways depend on iron and that their upregulation is consequently linked to an increased iron demand. In fact, ergosterol biosynthesis involves several iron-dependent enzymes such as Cyp51, Erg24, and CybE (37, 38), while adaptation to hypoxia involves upregulation of the tricarboxylic acid (TCA) cycle and oxidative phosphorylation, which depend on iron-sulfur clusters and heme as cofactors (39). In agreement, hypoxia was previously found to increase the cellular heme content of A. fumigatus (39). Consistent with the link between adaptation to hypoxia, iron and transcriptional regulation mediated by AtrR and SrbA, the growth defect of mutants lacking AtrR or SrbA during hypoxia was found to be largely cured by supplementation with high iron concentrations, such as 5 mM. An additional explanation might be the metabolic link between ergosterol and siderophore biosynthetic pathways, because mevalonic acid is not only an intermediate of ergosterol biosynthesis but also a precursor for biosynthesis of the extracellular siderophores FsC and TAFC (9). Consequently, adaptation to iron starvation is expected to require tuning of acetyl coenzyme A (acetyl-CoA) metabolism.

A highly interesting question is the mode of cooperation of SrbA and AtrA. Recently, a protein interacting with AtrR, termed nuclear coactivator of AtrR (NcaA), was identified by tandem affinity purification (40). In the underlying unbiased screen, SrbA was not found to interact with AtrR, which might indicate that SrbA and AtrR do not interact in solution. Possibly, these transcription factors interact only upon binding to DNA or indirectly. In this respect, it is important to note that single-gene ChIP indicated that deletion of the identified SrbA-binding sites in p*ftrA* did not affect promoter binding of AtrR. These data are in agreement with recently published data demonstrating that promoter occupation by both SrbA and AtrR is essential for transcriptional activation of *cyp51A* (26, 27).

In conclusion, this study elucidated a new function of AtrR and improved the understanding of the cooperation between AtrR and SrbA in transcriptional coordination of iron acquisition, adaptation to hypoxia, and biosynthesis of ergosterol and heme. The translational importance of the underlying metabolic pathways as well as their regulators, SrbA and AtrR, is evident from their essentiality for virulence and resistance against triazole drugs in A. fumigatus.

## MATERIALS AND METHODS

### Fungal strains and growth conditions.

The A. fumigatus strains used for phenotyping and transcriptional analysis were Afs35 (FGSC A1159), a derivative of the clinical isolate D141 lacking Ku70, termed the wild type (wt) here (41–43), and the derived Δ*atrR* (lacking AtrR), Δ*srbA* (lacking SrbA) (25), *atrR*^HA^ (AtrR C-terminally tagged with the hemagglutinin peptide) (24), *atrR*^p^*^hspA^* (overexpressing *atrR* under the control of the *hspA* promoter) (25), and *atrR*^p^*^hspA^ ΔsrbA* (lacking SrbA and overexpressing *atrR* under the control of the *hspA* promoter) mutants (25). Furthermore, A. fumigatus A1160^+^, a derivative of clinical isolate CEA17 lacking Ku80 (44), and the derived Δ*atrR* (lacking AtrR), Δ*srbA* (lacking SrbA), and Δ*sreA* (lacking SreA) mutants were used for phenotyping and for luciferase (*luc*) reporter assay-based promoter analyses (45). All used strains are listed in Table 1.

**TABLE 1:** Strains used in this study

Strain	Genotype	Source or reference
Afs35	Δ*ku70*::*loxP*	FGSC (41, 43, 44)
Δ*atrR* mutant	Afs35, Δ*atrR*::*ptrA*	25
Δ*srbA* mutant	Afs35, Δ*srbA::hph*	24
*atrR*^HA^ mutant	Afs35, *atrR*^HA^::*hph*	25
*atrR*^p^*^hspA^* mutant	Afs35, *ptrA*::p*hspA*-*atrR*	25
*atrR*^p^*^hspA^* Δ*srbA* mutant	Afs35, Δ*srbA ptrA:*:p*hspA-atrR*	25
A1160^+^	Δ*ku80*, *pyrG*^+^	52
Δ*atrR* mutant	A1160^+^, Δ*atrR*::*hph*	53
Δ*srbA* mutant	A1160^+^, Δ*srbA*::*hph*	22
Δ*sreA* mutant	A1160^+^, Δ*sreA*::*hph*	16
P1, P2, P3	A1160^+^, Δ*fcyB*∷p*ftrA* versions	This study
P4, P6, P7	A1160^+^, Δ*fcyB*∷p*ftrA* versions	This study
P4d^1^, P4d^3^, P4d^4^	A1160^+^, Δ*fcyB*∷p*ftrA* versions	This study
P4d^5^, P3m^2^	A1160^+^, Δ*fcyB*∷p*ftrA* versions	This study
P3m^4^, P3m^5^	A1160^+^, Δ*fcyB*∷p*ftrA* versions	This study
p*xylP*^289^ mutant	A1160^+^, Δ*fcyB*∷p*ftrA* version	This study
p*xylP*^289^ *ftrA*^210^ mutant	A1160^+^, Δ*fcyB*∷p*ftrA* version	This study
P3 Δ*sreA*	A1160^+^, Δ*sreA*::*hph*; Δ*fcyB*∷p*ftrA* version	This study
P3m^2^ Δ*sreA*	A1160^+^, Δ*sreA*::*hph*; Δ*fcyB*∷p*ftrA* version	This study
P3 Δ*atrR*	A1160^+^, Δ*atrR*::*hph*; Δ*fcyB*∷p*ftrA* version	This study
P3 Δ*srbA*	A1160^+^, Δ*srbA*::*hph*; Δ*fcyB*∷p*ftrA* version	This study
*srbA*^p^*^xylP^* Δ*atrR* mutant	Afs35, ΔatrR::*ptrA*; Δ*fcyB*∷*srbA*^p^*^xylP^*	This study
*srbA*^p^*^xylP^* Δ*srbA* mutant	Afs35, Δ*srbA::hph; ΔfcyB∷srbA*^p^*^xylP^*	This study
*srbA*^p^*^xylP^* mutant	Afs35, Δ*fcyB*∷*srbA*^p^*^xylP^*	This study

To generate conidia, A. fumigatus strains were grown at 37°C on Aspergillus complex medium (ACM) containing 2% (wt/vol) glucose, 0.2% (wt/vol) peptone, 0.1% (wt/vol) yeast extract, 0.1% (wt/vol) Casamino Acids, salt solution, and iron-free trace elements, as described in reference 46. For growth assays, strains were grown either on solid or in liquid AMM with 1% glucose as the carbon source and 20 mM glutamine as the nitrogen source. For iron-replete and high-iron conditions, FeSO_4_ was added as indicated. Iron-limiting conditions were achieved either by omitting iron or by the addition of the iron chelator bathophenanthroline disulfonic acid (BPS) to a final concentration of 200 μM. Plate growth assays were performed by point inoculating 1 × 10^4^ conidia on AMM plates incubated in normoxia (21% O_2_) or hypoxia (0.2% O_2_) at 37°C for 48 h. For biomass determination in liquid culture, 1 × 10^6^/mL conidia were inoculated in 100 mL AMM in 0.5-L Erlenmeyer flasks and shaken at 200 rpm at 37°C for 24 h.

### Generation of A. fumigatus *luc* reporter strains.

Oligonucleotides used to generate the plasmids with the desired genetic manipulation are listed in Table S1. The generated plasmids with promoter truncations, mutations, or deletions were introduced into the *fcyB* locus of A. fumigatus. Integration into the *fcyB* locus allows selection with 5-flucytosine without the need for a selection marker gene (31). To generate plasmids P1 to P7, four DNA fragments were amplified: (i) the plasmid backbone, including *fcyB*-flanking noncoding regions (NCR) amplified from template p*fcyB* (31); (ii) 1,326- to 154-bp long fragments of the intergenic region between *fetC* and *ftrA*, amplified from genomic A. fumigatus DNA; (iii) the codon-optimized Photinus pyralis *luc* gene (GenBank accession number KC677695) (47); and (iv) the *trpC* terminator sequence, amplified from P*gpdA*_LacZ_AtTrpCTerm_pJET1.2 (48). The generated fragments were then assembled using NEBuilder HiFi DNA assembly (New England Biolabs, Ipswich, MA, USA). Mutations were introduced into the plasmids by exchanging single base pairs using oligonucleotides shown in Table S1 and a Q5 site-directed mutagenesis kit (New England Biolabs, Ipswich, MA, USA). To generate plasmid p*xylP*^289^*ftrA*^210^, three DNA fragments were amplified: (i) a 210-bp fragment of p*ftrA*, amplified from P1; (ii) a 289-bp fragment of the previously reported p*xylP* promoter, amplified from Penicillium chrysogenum genomic DNA; and (iii) the *luc* gene with the *trpC* terminator amplified from P1. The fragments were assembled with the previously amplified plasmid backbone using NEBuilder HiFi DNA assembly (New England Biolabs, Ipswich, MA, USA). p*xylP*^289^ was generated by amplifying a 289-bp fragment of the previously reported p*xylP* promoter (32) from Penicillium chrysogenum genomic DNA and assembled with the previously amplified plasmid backbone and *luc* gene with the *trpC* terminator amplified from P1 using NEBuilder HiFi DNA assembly (New England Biolabs, Ipswich, MA, USA). After NotI-mediated linearization and purification with a Monarch PCR and DNA cleanup kit (New England Biolabs, Ipswich, MA, USA), the plasmids were transformed into A. fumigatus A1160^+^, Δ*atrR*, Δ*srbA*, and Δ*sreA* strains as described in reference 49. The potential transformants were then selected on AMM plates with 10 μg/mL flucytosine (TCI, Eschborn, Germany) and verified by Southern blotting (Fig. S6A).

10.1128/mbio.00757-23.1 TABLE S1 Oligonucleotides used in this study. Nucleotides in small letters are add-on sequences for cloning via NEB builder. Download Table S1, PDF file, 0.2 MB. Copyright © 2023 Yap et al. 2023 Yap et al. https://creativecommons.org/licenses/by/4.0/ This content is distributed under the terms of the Creative Commons Attribution 4.0 International license.

10.1128/mbio.00757-23.10 FIG S6 Scheme and confirmation of the introduction of the (A) p*ftrA luc* reporter and (B) *srbA*^p^*^xylP^* constructs in the *fcyB* locus of A. fumigatus. Genomic organization of the *fcyB* locus in wt and Δ*fcyB* strains. Generated genomic DNA digestion with EcoRV and XbaI, respectively, resulted in the expected fragment sizes detected by Southern blotting with digoxigenin (DIG)-labeled hybridization probes specific for the 5′ and 3′ flanking regions of *fcyB*. Download FIG S6, TIF file, 0.3 MB. Copyright © 2023 Yap et al. 2023 Yap et al. https://creativecommons.org/licenses/by/4.0/ This content is distributed under the terms of the Creative Commons Attribution 4.0 International license.

### Generation of A. fumigatus strains expressing *srbA* under the control of the *xylP* promoter.

Oligonucleotides used to generate the plasmid expressing *srbA* under the control of the *xylP* promoter (29, 30) listed in Table S1. The generated plasmid was introduced into the *fcyB* locus of A. fumigatus, which allows selection with 5-flucytosine without the need for a selection marker gene (31). To generate the plasmid, three DNA fragments were amplified: (i) the plasmid backbone including *fcyB* flanking noncoding regions (NCR) amplified from template p*fcyB* (31), (ii) p*xylP*, amplified from template pIG01 (30), (iii) the *srbA* coding sequence including 1000 bp of 3′-NCR, amplified from wt genomic DNA. The generated fragments were then assembled using NEBuilder HiFi DNA assembly (New England Biolabs, Ipswich, MA, USA). After NotI-mediated linearization and purification with a Monarch PCR and DNA cleanup kit (New England Biolabs, Ipswich, MA, USA), the plasmids were transformed into A. fumigatus wt, Δ*atrR*, and Δ*srbA* strains as described in reference 49. The potential transformants were then selected on AMM plates with 10 μg/mL flucytosine (TCI, Eschborn, Germany) and verified by Southern blotting (Fig. S6B).

### Siderophore production analysis.

For isolation and quantification of TAFC, 1 mL of culture supernatant was saturated with FeSO_4_ and mixed vigorously with 0.2 volume of chloroform. The chloroform phase containing TAFC was mixed with 1 volume water and 5 volumes diethyl ether. The TAFC content of the aqueous phase was measured photometrically at 440 nm using the molar extinction factor 2,996 M^−1^ cm^−1^ (14, 20). To determine the intracellular siderophore content, 50 mg of lyophilized mycelium was pulverized and suspended in 1 mL sodium phosphate buffer (50 mM, pH 7.5). The cell debris-free supernatant was then mixed with 0.25 volume of phenol-chloroform-isoamyl alcohol (PCI) (25:24:1). To the separated PCI phase containing the intracellular siderophore, 1 volume water and 5 volumes diethyl ether were added. The intracellular siderophore content of the aqueous phase was measured photometrically at 440 nm using the molar extinction factor 2,460 M^−1^ cm^−1^ (14, 50).

### Northern blot analysis.

Total RNA was isolated using TRI reagents (Sigma-Aldrich Corp., St. Louis, MO, USA) according to the manufacturer's manual. Formaldehyde (2.2 M) agarose gels were used to separate 10 μg of RNA and eventually blotted on Hybond-N+ membranes (Amersham Biosciences, Amersham, UK). Transcripts of interest were detected by hybridization with PCR-amplified digoxigenin (Roche Diagnostics GmbH, Mannheim, Germany)-labeled probes. The digoxigenin-labeled hybridization probes and the respective oligonucleotides used in this study are listed in Table S2.

### Determination of promoter activity.

Promoter activities of the *luc* reporter strains were determined by measuring bioluminescence after growth for 24 h at 37°C h in Lumitrac 96-well plates (Greiner Bio-One, Kremsmuenster, Austria). Therefore, 0.1 mL AMM per well was inoculated with 1.5 × 10^4^ spores. For Δ*atrR* and Δ*srbA* strains, 3 × 10^4^ spores were used to compensate for the reduced growth. For iron-replete conditions, 0.03 mM FeSO_4_ was added, and for iron-depleted conditions, iron was omitted. At 0.5 h after addition of 20 μL of 0.6 mM d-luciferin (Synchem UG & Co. KG, Felsberg/Altenburg, Germany) in phosphate-buffered saline (PBS), bioluminescence was measured at 580-80 nm using a spiral well scan employing a CLARIOstar Plus microplate reader (BMG Labtech, Offenburg, Germany). The background luminescence recorded from untransformed wt cells was subtracted. Three biological triplicates of each reporter strains were analyzed.

### ChIP analysis.

Chromatin immunoprecipitation was done as described in reference 25, with the following modifications. The strains P4, P4^m4^, P4^d1^, and P4^d3^ were grown in liquid AMM under iron-limiting conditions. Chromatin was fixed, sheared, and purified as described previously. After fixing, shearing, and purifying, the chromatin was incubated with anti-AtrR polyclonal antibody (25) at a dilution of 1:50 for 16 h on a nutator at 4°C. This sample was further incubated with 50 μL of protein A-Dynabeads (Life Technologies, Carlsbad, CA, USA) for 8 h at 4°C. Real-time PCR of DNA that had been subjected to ChIP was performed as described in reference 51 with the following modifications: Percentage enrichment was calculated by determining the enrichment of the *ftrA* promoter driving *luc* compared to the native *ftrA* promoter in the ChIP DNA samples, after normalizing difference in primer efficiency of the two primer sets using input DNA. The oligonucleotide primer pairs used to analyze promoter enrichment are listed in Table S3.

10.1128/mbio.00757-23.3 TABLE S3 Oligonucleotide primer pairs used to analyze promoter enrichment. Download Table S3, PDF file, 0.2 MB. Copyright © 2023 Yap et al. 2023 Yap et al. https://creativecommons.org/licenses/by/4.0/ This content is distributed under the terms of the Creative Commons Attribution 4.0 International license.
