# Supplementary material for: Regulation of High-Affinity Iron Acquisition, Including Acquisition Mediated by the Iron Permease FtrA, Is Coordinated by AtrR, SrbA, and SreA in Aspergillus fumigatus
Source: mBio. 2023 Apr 24;14(3):e00757-23. doi: 10.1128/mbio.00757-23 (PMC10294635; doi:10.1128/mbio.00757-23)
Supplement: TABLE S2 [file mbio.00757-23-s0007.pdf]

Probe	Gene	Primer sequence
atrR	AFUA_5G02690	AGAAGCGTCTTGCTGACC
	Zn_2Cys_6 TF	TCAGACCGAAGCCAG AAC
cdr1B	AFUA_1G14330	ACCAAGGAGGATTCGACG
	ABC transporter	AGCGGTTGTCTGACGTTC
cyp51A	AFUA_4G06890	CGAACAGAACCGCCAATG
	14-alpha sterol demethylase	AAGTGTTCGTCGCTGAGG
erg25A	AFUA_8G02440	AGATAATCCCACCACCGC
	C-4 methyl sterol oxidase	CTCCTGGAACTTACTGGG
erg3a	AFUA_2G00320	ATGCGCTTCTTCTGCCAG
	Sterol Δ5,6-desaturase	AACTTGCCGCTGGATCTC
ftrA	AFUA_5G03810	GGGACAAGAGCAAGATGC
	ferric iron transporter	CCCAGTAGAGGATGCAAG
hapX	AFUA_5G03920	TCGGTGGAAAGAAGTGCC
	bZIP transcription factor	CGAGTCCGTTTGGGTATC
hemF	AFUA_1G07480	AACGCTCCGATGAACACC
	Coproporphyrinogen III oxidase	GGTCTCTTGAGGGTTCTG
mirB	AFUA_3G03640	AAGCCGAGAAAAAGGGGG
	siderophore transporter	AACCCAGATGAAGCCCAG
sidA	AFUA_2G07680	AACTACCTCCACCAGAAG
	N5-ornithine-monooxygenase	GAACGGCAATGTTGTAAG
srbA	AFUA_2G01260	ATGGGATGACGAGCAAGC
	SREBP TF	TCAAGTACCATGAGCCCG
srbB	AFUA_4G03460	AACAGACCTGATGCCTCC
	SREBP	TCTCCTTGTTATGCCGCC
