# Supplementary figures and images for: Regulation of High-Affinity Iron Acquisition, Including Acquisition Mediated by the Iron Permease FtrA, Is Coordinated by AtrR, SrbA, and SreA in Aspergillus fumigatus
Source: mBio. 2023 Apr 24;14(3):e00757-23. doi: 10.1128/mbio.00757-23 (PMC10294635; doi:10.1128/mbio.00757-23)

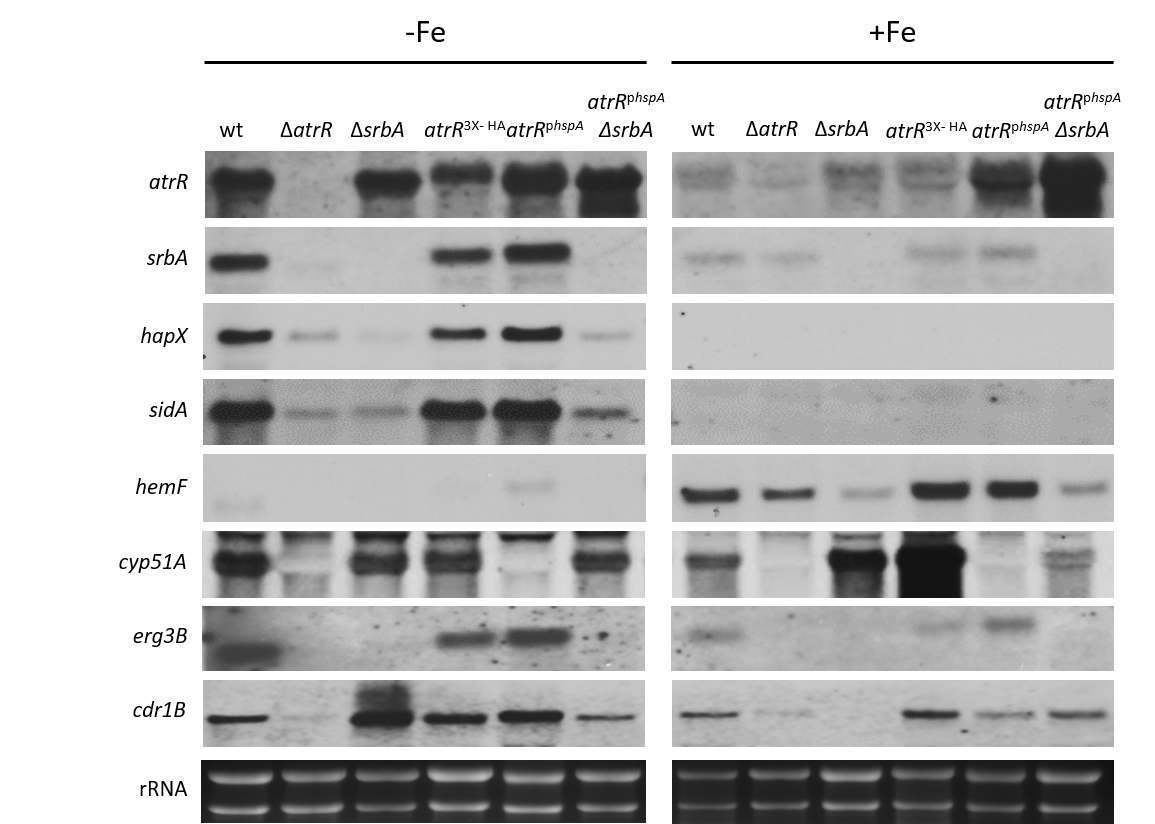

Supplement: FIG S1 [file mbio.00757-23-s0001.tif]

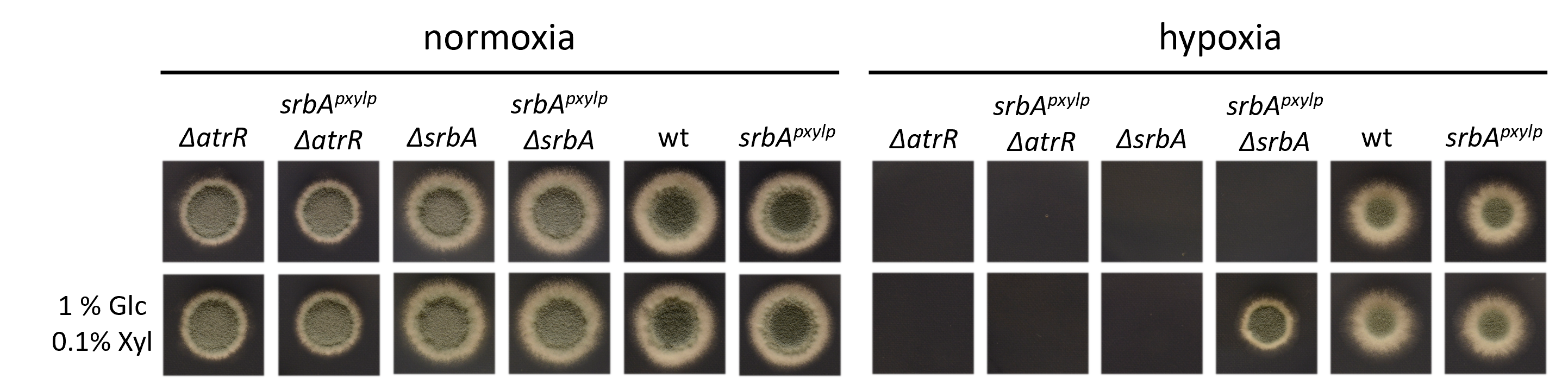

Supplement: FIG S2 [file mbio.00757-23-s0002.tif]

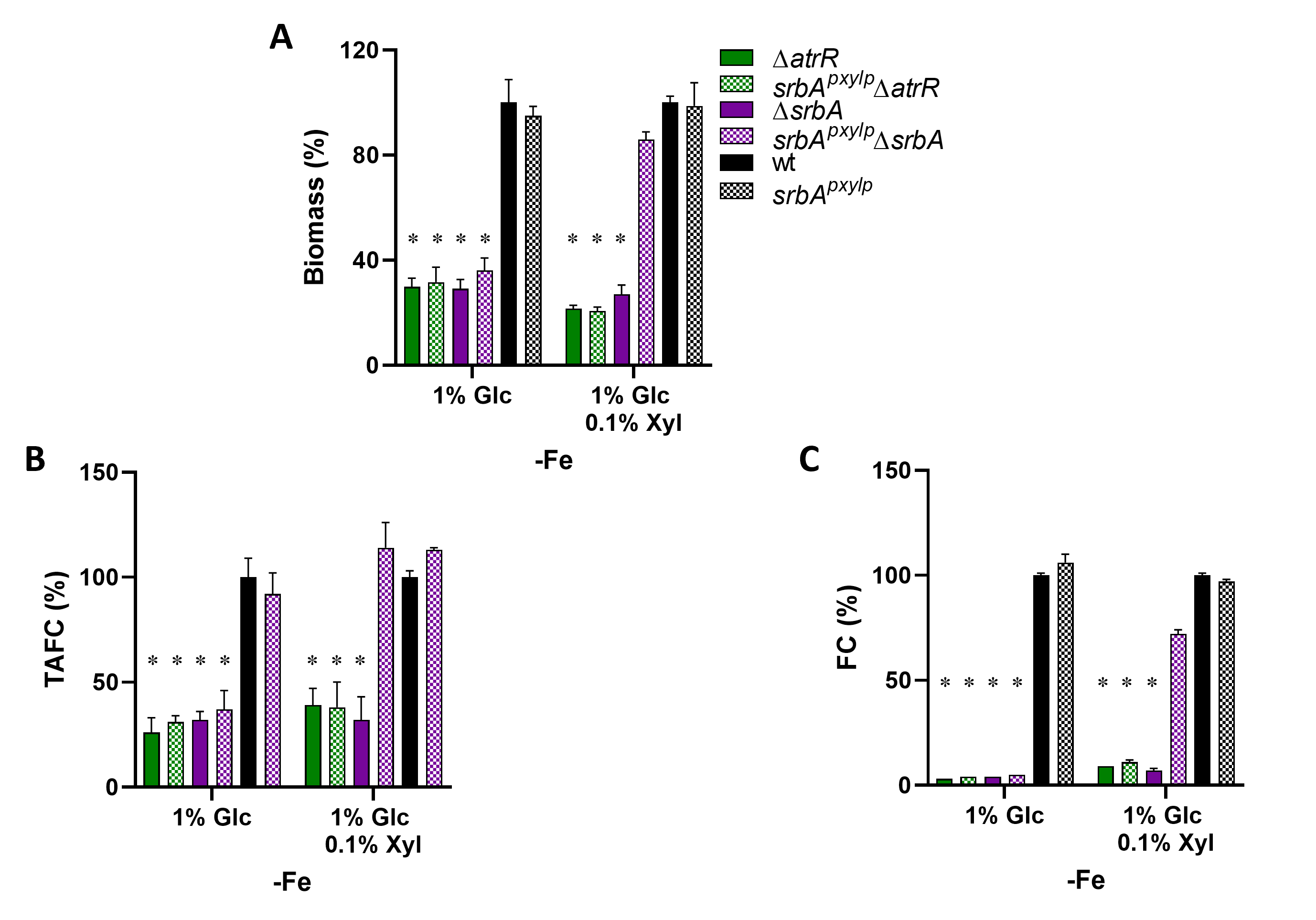

Supplement: FIG S3 [file mbio.00757-23-s0003.tif]

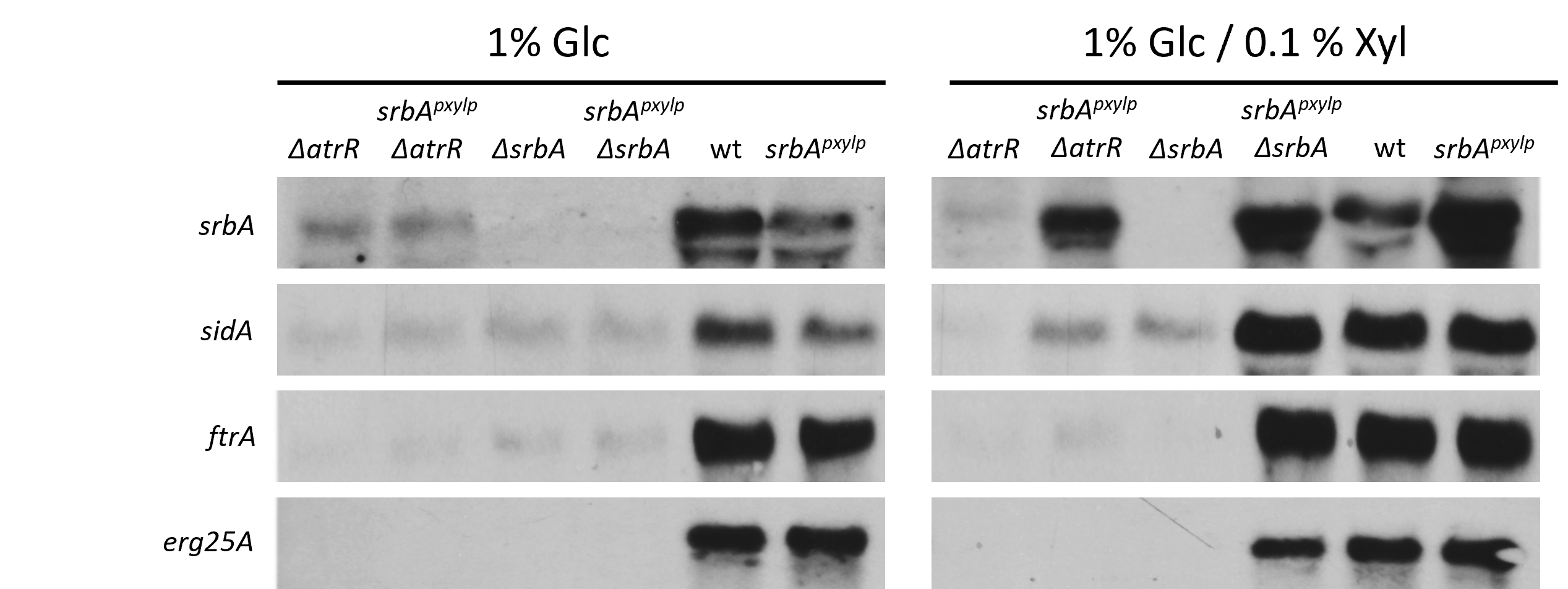

Supplement: FIG S4 [file mbio.00757-23-s0004.tif]

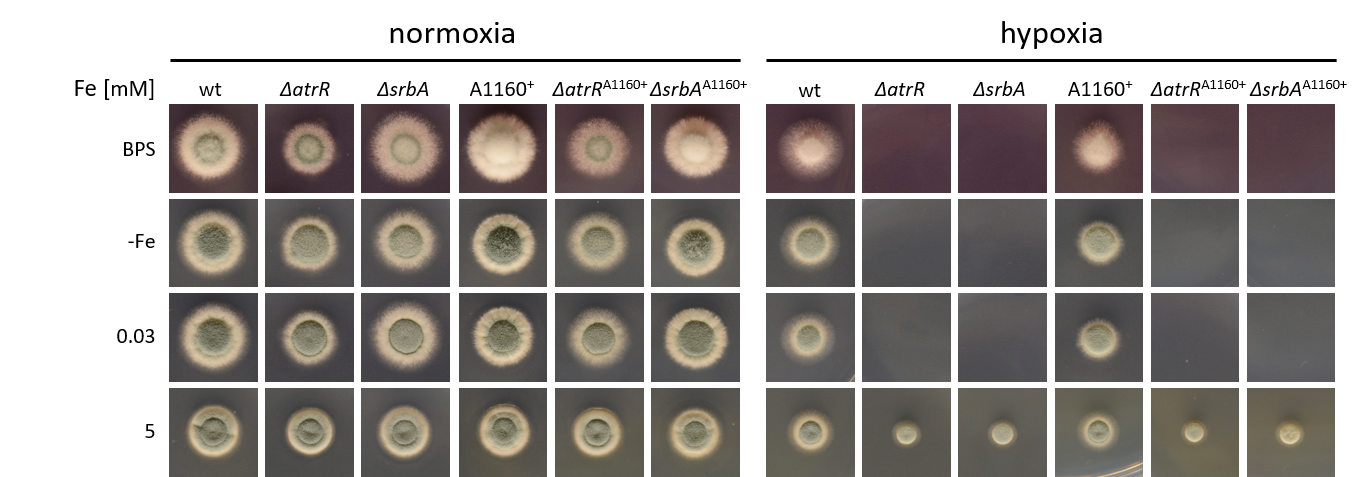

Supplement: FIG S5 [file mbio.00757-23-s0005.tif]

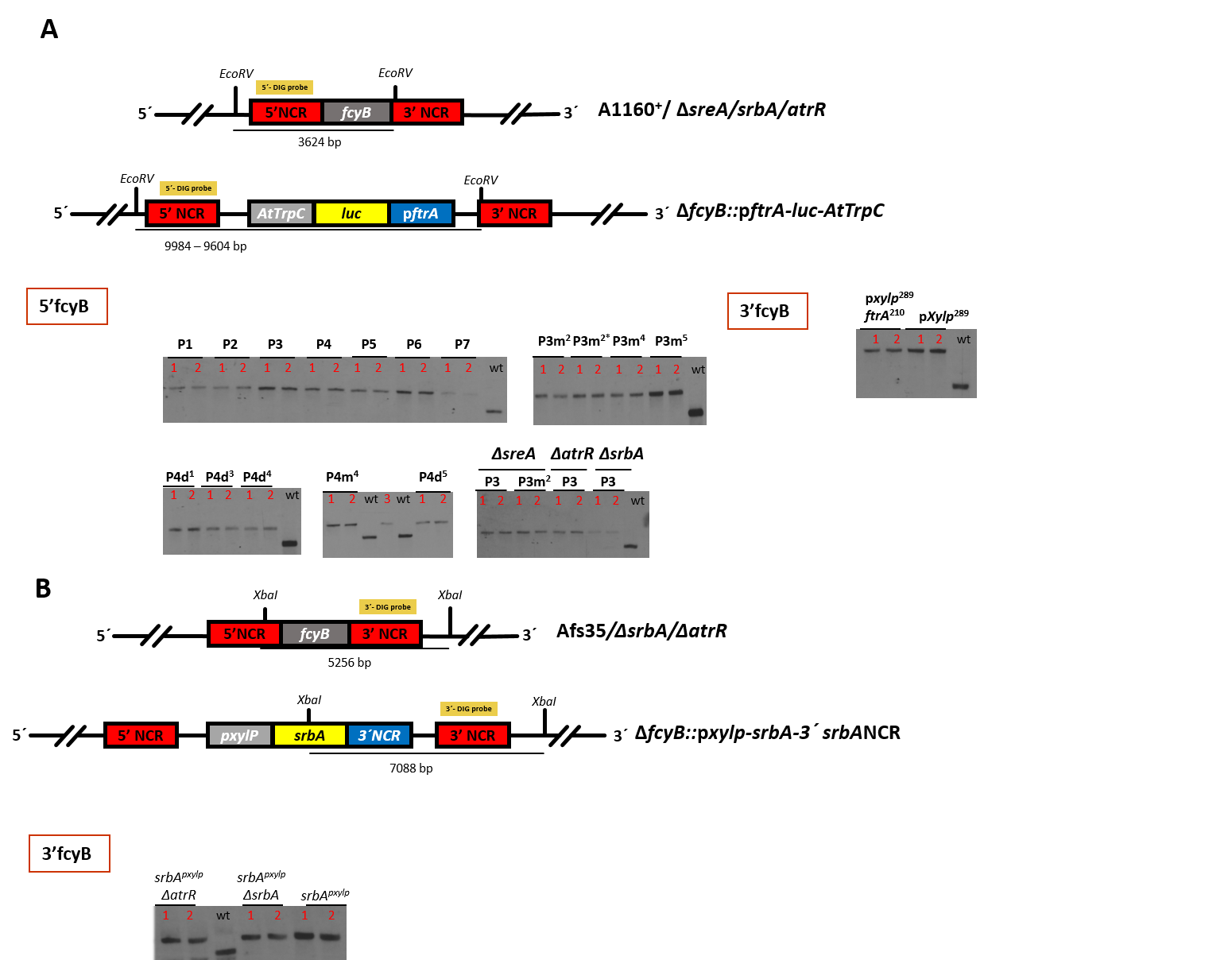

Supplement: FIG S6 [file mbio.00757-23-s0006.tif]
