# Supplementary material for: Regulation of High-Affinity Iron Acquisition, Including Acquisition Mediated by the Iron Permease FtrA, Is Coordinated by AtrR, SrbA, and SreA in Aspergillus fumigatus
Source: mBio. 2023 Apr 24;14(3):e00757-23. doi: 10.1128/mbio.00757-23 (PMC10294635; doi:10.1128/mbio.00757-23)
Supplement: TABLE S4 [file mbio.00757-23-s0008.pdf]

Experiment A

	-Fe			+Fe		
	1	2	3	1	2	3
P1	50745	47857	44766,5	7845	8252	8401
P2	77745	78909	73927	14669	13819	13401
P3	96592	84149	86201	12754	13981	13109
P4	80593	84619	85757	13417	13414	12984
P6	5663	5052	4459	1755	1819	1838
P7	3449	3646	3658	1468	1618	1592
P4d^{1}	7174	6461	7072	3124	3492	3352
P4d^{1}	8511	7088	7139	2786	3129	2973
P4d^{3}	27531	28773	27832	7627	6719	7379
P4d^{3}	20246	19583	17203	5342	5402	5511
P4d^{4}	17079	15783	15707	2016	1813	2258
P4d^{4}	19222	18176	17681	3092	2275	2050
P4	64883	77499	76590	16770	16885	18693

Experiment B

	-Fe			+Fe		
	1	2	3	1	2	3
p*xylp*^{289}*ftrA*^{210}	108014	99803	99305	15709	16919	14574
p*xylp*^{289}	14859	13481	14226	9297	8670	10680
P3	99912	99070	98712	32093	33190	35009

Experiment C

	-Fe			+Fe		
	1	2	3	1	2	3
P3m^{2}	104558	110908	115672	83962	96322	108145
P3m2*	87816	85832	89764	63895	73915	66121
P3 ΔsreA	111302	110697	109366	107842	113224	135824
P3m^{2}ΔsreA	115053	108814	113271	99480	86533	101648
P3	104558	110908	115672	43501	46115	41601
P3m^{4}	122057	101367	101367	39879	40080	40967
P3m^{4}	139669	139530	122195	44509	43996	37374
P3m^{5}	97366	89923	84688	13853	11279	9758
P3m^{5}	92084	86138	80561	11358	9632	10093
P3	193641	174174	164370	41924	39627	41997
P3 Δ*srbA*	9617	9533	10274	980	994	923
P3 Δ*atrR*	6965	6625	7344	1993	1890	1950
P4m^{4}	115810	108004	96210	21528	23160	22450
P4	156679	162050	156533	36615	31058	29115
