# Supplementary material for: Regulation of High-Affinity Iron Acquisition, Including Acquisition Mediated by the Iron Permease FtrA, Is Coordinated by AtrR, SrbA, and SreA in Aspergillus fumigatus
Source: mBio. 2023 Apr 24;14(3):e00757-23. doi: 10.1128/mbio.00757-23 (PMC10294635; doi:10.1128/mbio.00757-23)
Supplement: TABLE S1 [file mbio.00757-23-s0010.pdf]

Plasmid	Description	Primer Sequence
p1	plasmid backbone	AAACAGCTATGACCATGATTAC<br>TGTGAAATTGTTATCCGCTC
	p*ftrA*	cgtcctccatCTTGTGAGTCGCGAGGGAG<br>gagcggataacaatttcacaGGCACCTCCGAAAGACGATG
	luc	aatcatggtcatagctgtttAAGCTTGAGTGAGGGTTG<br>gaaatcactgctgccatggCTAGACGGCGATCTTGCCGCC
	trpC terminator	aatcatggtcatagctgtttAAGCTTGAGTGAGGGTTG<br>ggcggcaagatcgccgtctagCCATGGCAGCAGTGATTTC
p2		gagcggataacaatttcacaCTACTGGGGGGAAGAACG<br>cgtcctccatCTTGTGAGTCGCGAGGGAG
p3		gagcggataacaatttcacaAGGACCGTACGATCCCAATTG<br>cgtcctccatCTTGTGAGTCGCGAGGGAG
p4		gagcggataacaatttcacaCATCCAGGACAGTTCTCGC<br>cgtcctccatCTTGTGAGTCGCGAGGGAG
p6		gagcggataacaatttcacaTGCTGTGGCCACACAATTC<br>cgtcctccatCTTGTGAGTCGCGAGGGAG
p7		gagcggataacaatttcaca<br>TCTTTCAATCTTTATCGATCAGTCAC<br>cgtcctccatCTTGTGAGTCGCGAGGGAG
p3m^{2}	Site directed mutagenesis on p3	TCATCGAATCGGGGGGCCTTTTCC<br>TTTCGAAAAATGGCGCAAGAACGGAAAC
p3m^{2*}	Site directed mutagenesis on p3	TCCGAATCGGGGGGCCTTTTCC<br>AAAATGGCGCAAGAACGGAAAC
p3m^{4}	Site directed mutagenesis on p3	TTTTCCCCTCGTGACTATACGCATTTTTTATC<br>GGCCCCGTAATTATCTGATTGATAAAAATGGC
p3m^{5}	Site directed mutagenesis on p3	AAAATATGCTGTGGCCACACAAT<br>AGAATTGATTGATTGATAAAAAATGCGTATAG
p4d^{1}	Site directed mutagenesis on p4	TGCTGTGGCCACACAATTC<br>ACGGAAACCTCGGCAACA
p4d^{3}	Site directed mutagenesis on p4	GACTATACGCATTTTTTATCAATCAATCAATC<br>ACGGAAACCTCGGCAACA
p4d^{4}	Site directed mutagenesis on p4	TGCTGTGGCCACACAATTC<br>ACGAGGGGAAAAGGCCCC
p4d^{5}	Site directed mutagenesis on p4	CGTGACTATACGCATTTTTTATC<br>ATTATCTGATTGATAAAAATGGC
p4m^{4}	Site directed mutagenesis on p4	TTTTCCCCTCGTGACTATACGCATTTTTTATC<br>GGCCCCGTAATTATCTGATTGATAAAAATGGC
p*xylP*^{289}*ftrA*^{210}	p*ftrA*	gcagagaggactttattcTGTATGGCCTGATTGATTGATTG<br>gagcggataacaatttcacaCATCCAGGACAGTTCTCGC
	minprom	atgttcttggcgtcctccatGGTTGGTTCTTCGAGTCG<br>agcggataacaatttcacaCAGAATAAAGTCCTCTCTGC
	Luc+ trpC terminator	catcgactcgaagaaccaaccATGGAGGACGCCAAGAAC<br>aatcatggtcatagctgtttAAGCTTGAGTGAGGGTTG
p*xylP*^{289}		agcggataacaatttcacaCAGAATAAAGTCCTCTCTGC<br>atgttcttggcgtcctccatGGTTGGTTCTTCGAGTCG
p*srbA*^{pxlyp}	*pxylp*	agcggataacaatttcacaGCACTGATGCGAGCAACAG<br>aatgccgggggtggacatGGTTGGTTCTTCGAGTCG
	*srbA*_3´NCR	cgactcgaagaaccaaccATGTCCACCCCCGGCATT<br>aatcatggtcatagctgtttGTCAGGGAAGAGCAAAGACT
