# Supplementary material for: Regulation of High-Affinity Iron Acquisition, Including Acquisition Mediated by the Iron Permease FtrA, Is Coordinated by AtrR, SrbA, and SreA in Aspergillus fumigatus
Source: mBio. 2023 Apr 24;14(3):e00757-23. doi: 10.1128/mbio.00757-23 (PMC10294635; doi:10.1128/mbio.00757-23)
Supplement: TABLE S3 [file mbio.00757-23-s0009.pdf]

Name	Description	Primer Sequence
ftrA-ChiPF	Native *ftrA* promoter	ATCCAGGACAGTTCTCGCT
ftrA-ChipR		AGCAAATACGTCTTTTGCCA
Luc-ChiPF	Hybrid *ftrA* promoter	ATCCAGGACAGTTCTCGCT
Luc-ChipR		TTCTTGGCGTCCTCCAT
